# Approximate Uncertainty Modeling in Risk Analysis with Vine Copulas

**DOI:** 10.1111/risa.12471

**Published:** 2015-09-02

**Authors:** Tim Bedford, Alireza Daneshkhah, Kevin J. Wilson

**Affiliations:** ^1^ Department of Management ScienceUniversity of StrathclydeGlasgowUK; ^2^ Cranfield Water Science InstituteCranfield UniversityBedfordUK

**Keywords:** Copula, entropy, information, risk modeling, vine

## Abstract

Many applications of risk analysis require us to jointly model multiple uncertain quantities. Bayesian networks and copulas are two common approaches to modeling joint uncertainties with probability distributions. This article focuses on new methodologies for copulas by developing work of Cooke, Bedford, Kurowica, and others on vines as a way of constructing higher dimensional distributions that do not suffer from some of the restrictions of alternatives such as the multivariate Gaussian copula. The article provides a fundamental approximation result, demonstrating that we can approximate any density as closely as we like using vines. It further operationalizes this result by showing how minimum information copulas can be used to provide parametric classes of copulas that have such good levels of approximation. We extend previous approaches using vines by considering nonconstant conditional dependencies, which are particularly relevant in financial risk modeling. We discuss how such models may be quantified, in terms of expert judgment or by fitting data, and illustrate the approach by modeling two financial data sets.

## 1. INTRODUCTION

Many areas of applied risk analysis require us to model multiple uncertainties using multivariate distributions. For some decision support settings, it is common to use discrete models such as Bayesian networks. In other settings, particularly when modeling financial data or carrying out uncertainty analysis, it is necessary to have models of multivariate continuous random variables. Dependency modeling is therefore an area of great interest for a whole range of risk analysis applications.

There is a growing literature on the use of copulas to model dependencies (see, e.g., surveys by Nelsen and^1^, ^2^ Joe^3^). Copulas have found application in a number of areas, including combining expert opinion and stochastic simulation.^4^, ^5^, ^6^, ^7^, ^8^, ^9^ A copula is a joint distribution on the unit square (or more generally on the unit *n*‐cube) with uniform marginal distributions. Under reasonable conditions, we can uniquely specify a joint distribution for *n* random variables by specifying the univariate distribution for each variable, and, in addition, specifying the copula. This is because we can simply transform each variable by its own distribution function (sometimes called its quantile function) to ensure that the transformed variable has a uniform distribution, so that the joint distribution function *F* can be written:

(1) $$F\left(x_{1},...,x_{n}\right)=C\left(F_{1}\left(x_{1}\right),...,F_{n}\left(x_{n}\right)\right),$$

where *C* is a copula distribution function, and $F_{1},...,F_{n}$ are the univariate, or marginal, distribution functions. We can use this formula constructively: given a copula *C* and marginals $F_{1},...,F_{n}$ we can *define F* in this way. A special case is that of the “Gaussian copula,” obtained from the Gaussian joint distribution and parameterized by the correlation matrix. Use of the Gaussian copula to construct joint distributions is equivalent to the NORTA method (normal to anything).^10^

The use of a copula to model dependency is simply a translation of one difficult problem into another: instead of the difficulty of specifying the full joint distribution we have the difficulty of specifying the copula. The main advantage is the technical one that copulas are normalized to have support on the unit square and uniform marginals. As many authors restrict the copulas to a particular parametric class (Gaussian, multivariate *t*, etc.) the potential flexibility of the copula approach is not realized in practice. The approach used in this article, by contrast, allows a lot of flexibility in copula specification. It utilizes a graphical model, called a vine, to systematically specify how two‐dimensional copulas are stacked together to produce an *n*‐dimensional copula.

The main objectives of this article are to show that a vine structure can be used to approximate any given multivariate copula to any required degree of approximation, and to show how this can be operationalized for use in practical situations involving uncertain risks. The standing technical assumptions we make are that the multivariate copula density *f* under study is continuous and is nonzero. No other assumptions are needed. We illustrate this by modeling a data set of Norwegian financial data that was previously analyzed in Aas *et al*.^11^ We extend the modeling approach used by Aas *et al*.^11^ by considering the possibility of nonconstant conditional dependencies within the vine structure.

Since vines demonstrate high flexibility and advantages in constructing multivariate distributions, they have recently been used to describe the inner‐dependence structure and build the joint distribution of portfolio returns. As coherent measures of risk, value at risk (VaR) and conditional value at risk (CVaR), which are greatly affected by the tail distribution of risk factors, have been widely used to optimize portfolios and measure their risk. Deng *et al*.^12^ used extreme value theory to model the tails of the innovation of each asset return and estimate risk of assets. The dependence structure between innovations of asset returns can be represented by a vine. This vine is useful to model both the influence of portfolio dimensions and the differences of tail dependence between assets. As expected, the optimal portfolio is better via a vine than that via a Student copula model (see also Ref. 11 for similar study). We illustrate that the minimum information vine can outperform the standard multivariate copula model and specific parametric vines.^13^

Our constructive approach involves the use of minimum information copulas that can be specified to any required degree of precision based on the data available. We prove rigorously that good approximation “locally” guarantees good approximation globally. Finally, we discuss rules of thumb that could be used to apply this in practice. In particular, we discuss vine structure. A vine structure imposes no restrictions on the underlying joint probability distribution it represents (as opposed to the situation for Bayesian networks, for example). However, this does not mean that we should ignore the question about which vine structure is most appropriate, for some structures allow the use of less complex conditional copulas than others. Conversely, if we only allow certain families of copulas, then one vine structure might fit better than another.

## 2. VINE CONSTRUCTIONS FOR MULTIVARIATE DEPENDENCE

A copula is a multivariate distribution function with standard uniform marginal distributions. Using Equation (1), a copula can be used, in conjunction with the marginal distributions, to model any multivariate distribution. However, apart from the multivariate Gaussian, Student, and the exchangeable multivariate Archimedean copulas, the set of higher dimensional copulas proposed in the literature is limited and is not rich enough to model all possible mutual dependencies among the *n* variates (see Kurowicka and Cooke^14^ for details of these copulas). Hence, it is necessary to consider more flexible constructions.

A structure, here denoted the *pair‐copula construction* or *vine*, allows for the free specification of (at least) n(n−1)/2 copulas between *n* variables. (Note that n(n−1)/2 is the number of entries above the diagonal of an n×n correlation matrix—though these are algebraically related so not completely free variables.) This structure was originally proposed by Joe,^3^ and reformulated and discussed in detail by Bedford and Cooke,^13^, ^15^ who considered simulation, information properties, and the relationship to the multivariate normal distribution but who considered a more general method called a Cantor tree construction.

**Figure 1:**
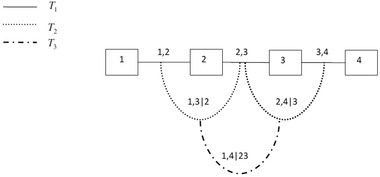
A regular vine with four elements.

Kurowicka and Cooke^14^ (Chapters 4, 6–9) consider simulation issues and Aas *et al*.^11^ look at inference. Excellent overviews of vines are given in Refs. 16 and 17. The modeling scheme is based on a decomposition of a multivariate density into a set of bivariate copulas. The way these copulas are built up to give the overall joint distribution is determined through a structure called a vine, and can be easily visualized. A vine on *n* variables is a nested set of trees, where the edges of the tree *j* are the nodes of the tree j+1 (for j=1,...,n−2), and each tree has the maximum number of edges. For example, Fig. 1 shows a vine with four variables, which consists of three trees ($T_{1},T_{2},T_{3}$) with 3, 2, and 1 edges, respectively. A *regular vine* on *n* variables is a vine in which two edges in tree *j* are joined by an edge in tree j+1 only if these edges share a common node, for j=1,...,n−2. There are n(n−1)/2 edges in a regular vine on *n* variables. The formal definition is as follows.

DEFINITION 1 **(Vine, regular vine)** V is a vine on *n* elements if

(1) $V=(T_{1},...,T_{n-1})$.
(2) *T*_1_ is a connected tree with nodes $N_{1}=\left\{1,...,n\right\}$ and edges *E*_1_; for i=2,...,n−1, $T_{i}$ is a connected tree with nodes $N_{i}=E_{i-1}$.
V is a regular vine on *n* elements if additionally the proximity condition holds.
(3) For i=2,...,n−1, if *a* and *b* are nodes of $T_{i}$ connected by an edge in $T_{i}$, where $a=\{a_{1},a_{2}\}$, $b=\{b_{1},b_{2}\}$, then exactly one of the $a_{i}$ equals one of the $b_{i}$.

One of the simplest regular vines is shown in Fig. 1—this structure is called a *D*‐vine; see Kurowicka and Cooke,^14^ p. 93. Here, *T*_1_ is the tree consisting of the straight edges between the numbered nodes, *T*_2_ is the tree consisting of the curved edges that join the straight edges in *T*_1_, and so on.

For a regular vine, each edge of *T*_1_ is labeled by two numbers from {1,...,n}. If we take two edges of *T*_1_, for example, 12 and 23, which are nodes joined by an edge in *T*_2_, then of the numbers labeling these edges one is common to both (2), and they both have one unique number (1,3, respectively). The common number(s) will be called the *conditioning* set $D_{e}$ for that edge *e* (in this example, the conditioning set is simply {2}) and the other numbers will be called the *conditioned set* (in this example, {1,3}). For a regular vine, the conditioned set always contains two elements.

We associate a vine distribution to a vine by specifying a copula to each edge of *T*_1_ and a family of conditional copulas for the conditional variables given the conditioning variables, as shown by the following result of Bedford and Cooke.^15^

THEOREM 1 Let $V=(T_{1},...,T_{n-1})$ be a regular vine on *n* elements. For each edge $e\left(j,k\right)\in T_{i},i=1,...,n-1$ with conditioned set {j,k} and conditioning set $D_{e}$, let the conditional copula and copula density be $C_{jk|De}$ and $c_{jk|De}$, respectively. Let the marginal distributions $F_{i}$ with densities $f_{i},i=1,...,n$ be given. Then, the vine‐dependent distribution is uniquely determined and has a density given by:

(2) $$\begin{array}{ccc} & & f(x_{1},...,x_{n})=\\ & & \prod_{i=1}^{n}f\left(x_{i}\right)\prod_{j=1}^{n-1}\prod_{e\left(j,k\right)\in E_{i}}c_{jk|D_{e}}\left(F_{j|D_{e}\left(x_{j}\right)},F_{k|D_{e}\left(x_{k}\right)}\right). \end{array}$$

Note that we use $c_{jk|De}$ here to be a conditional copula density and not the more usual conditional bivariate cumulative distribution function (cdf) (which is not a copula). The existence of regular vine distributions is discussed in detail by Bedford and Cooke.^13^

The density decomposition associated with four random variables $X=(X_{1},...,X_{4})$ with a joint density function $f(x_{1},...,x_{4})$ satisfying a copula‐vine structure shown in Fig. 1 with the marginal densities $f_{1},...,f_{4}$ is:

$$\begin{array}{ccc} f_{1234}\left(x_{1},...,x_{4}\right) & = & \prod_{i=1}^{4}f\left(x_{i}\right)\times c_{12}\left(F\left(x_{1}\right),F\left(x_{2}\right)\right)\\ & & c_{23}\left(F\left(x_{2}\right),F\left(x_{3}\right)\right)c_{34}\left(F\left(x_{3}\right),F\left(x_{4}\right)\right)\times \end{array}$$

(3) $$\begin{array}{ccc} & & c_{13|2}\left(F\left(x_{1}|x_{2}\right),F\left(x_{3}|x_{2}\right)\right)c_{24|3}\left(F(x_{2}|x_{3}),\right.\\ & & \left.F(x_{4}|x_{3})\right)\times c_{14|23}\left(F\left(x_{1}|x_{2},x_{3}\right),F\left(x_{4}|x_{2},x_{3}\right)\right). \end{array}$$

Note that in the special case of a joint normal distribution, we would use the normal copula everywhere in the above expression and the conditional copulas would be constant (i.e., not depend on the conditioning variable). This means that the joint normal structure is specified by n(n−1)/2 (conditional) correlation values, which are algebraically free between −1 and +1 (unlike the values in a correlation matrix). See Bedford and Cooke^13^ for more details.

Theorem 1 gives us a constructive approach to build a multivariate distribution given a vine structure: if we choose marginal densities and copulas, then this will give us a multivariate density. Hence, vines can be used to model general multivariate densities. However, in practice we have to use copulas from a convenient class, and this class should ideally be one that allows us to approximate any copula to an arbitrary degree. In the following sections, we address this issue in more detail. By having this class of copulas, we can approximate any multivariate distribution using any vine structure.

Unlike the situation with Bayesian networks, where not all structures can be used to model a given distribution, the theorem shows that, in principle, any vine structure may be used to model a given distribution. However, when specific families of copulas are used some vine structures work better than others. That is, given a family of copulas, some vine structures give a better degree of approximation than others. We shall return to this point later.

Much work has been done to operationalize the use of vines for modeling multivariate data sets^11^, ^16^, ^18^, ^19^ and an R package “VineCopula” has been developed to implement the approaches in this work (http://cran.r‐project.org/web/packages/VineCopula/index.html).

It is worth stressing that the flexibility of vines gives us the potential to capture any fine‐grain structure within a multivariate distribution. A key aspect that cannot be modeled by Bayesian networks is that of conditional dependence. Bayesian networks are built around the concept of conditional *independence*—arrows from a parent node to two child nodes means that the child variables are conditionally, independent given the parent variable. Of course, unconditionally these two child nodes are dependent. However, different models of conditional dependence are not available as building blocks in Bayesian networks.

Multivariate Gaussian copulas do allow for a specification of conditional dependence, but do not allow that dependence to change: in a multivariate normal distribution, the conditional correlation of two variables given a third may be nonzero but is always constant. Our approach allows the explicit modeling of nonconstant conditional dependence, as we illustrate with a simple example.

The deeper a bivariate copula is in the vine hierarchy, the more variables will be conditioned on. If the conditional dependencies are neglected, then vines are a direct method to build flexible multivariate models using bivariate copulas as building blocks. Acar *et al*.^20^ argue that ignoring conditional dependencies (the so‐called simplifying assumption) can lead to reasonably precise approximations of the underlying copula (as claimed by Ref. 21), but this can in general be misleading. They develop an approach to condition parametric bivariate copulas on a single scalar variable. Stoeber *et al*.^22^ repeated this concern, after studying several examples, and felt the assumption of an absolutely continuous vine is sometimes too strong. The latter assumption is used to make the vine models tractable for inference and model selection. Lopez‐Paz *et al*.^23^ also reported that the simplifying assumption can lead to oversimplified estimates in practice. They then extended the work of Acar *et al*. by developing a method for estimation of fully conditional vines using a Gaussian Process.

### 2.1. Example

We consider an example involving nonconstant conditional correlations. Suppose we have three unknown quantities, $X_{1},X_{2},X_{3}$, for which we wish to specify a joint distribution. Marginally each variable is normally distributed, $X_{i}∼N\left(m_{i},s_{i}^{2}\right)$, for i=1,2,3, and $X_{i}$ is not independent of $X_{j}$ for i≠j. We can represent the joint distribution between $X_{1},X_{2},X_{3}$ using a D‐vine in three dimensions. That is, specify a copula between $X_{1},X_{2}$, one between $X_{2},X_{3}$, and then a conditional copula between $X_{1},X_{3}|X_{2}$.

In each case, we choose a bivariate Gaussian copula. This takes the form, for $U_{i}=F\left(x_{i}\right)$,

$$C\left(u_{i},u_{j}\right)=\Phi_{\rho }\left(\Phi^{-1}\left(u_{i}\right),\Phi^{-1}\left(u_{j}\right)\right),$$

where Φ(·) is the cdf of the standard Normal distribution and $\Phi_{\rho }\left(\cdot ,\cdot \right)$ is the cdf of the standard bivariate Normal distribution with correlation ρ. Suppose that the correlations in the first tree of the vine are specified as ρ_12_ and ρ_23_ for the marginal copulas between $X_{1},X_{2}$ and $X_{2},X_{3}$, respectively.

If we were to specify a constant correlation between $X_{1},X_{3}|X_{2}$ then the resulting distribution of $X_{1},X_{2},X_{3}$ could be modeled using the Gaussian copula. However, let us suppose that the correlation between $X_{1},X_{3}|X_{2}$ is not constant but rather

$$\begin{array}{ccc} \rho_{X_{1},X_{3}|X_{2}\in \left(0:0.33\right)} & = & 1,\rho_{X_{1},X_{3}|X_{2}\in \left(0.33:0.67\right)}=0,\\ & & \rho_{X_{1},X_{3}|X_{2}\in \left(0.67:1\right)}=-1, \end{array}$$

so that there is a positive linear relationship between the variables for $U_{2}=F_{2}\left(X_{2}\right)$ in $\left(0,\frac{1}{3}\right)$, they are *uncorrelated* for *U*_2_ between $\left(\frac{1}{3},\frac{2}{3}\right)$, and there is a negative linear relationship between them in $\left(\frac{2}{3},1\right)$.

We can divide the support of *X*_2_, via *U*_2_, into intervals. Then we can define a Gaussian copula within each interval. Suppose that numerical values for the required means and standard deviations are $m_{1}=0.5,m_{2}=1,m_{3}=-1$, and $s_{1}=s_{2}=s_{3}=2$ and the correlations between $X_{1},X_{2}$ and $X_{2},X_{3}$ are $\rho_{12}=0.75$, $\rho_{23}=-0.75$, respectively. This fully specifies the vine.

**Figure 2:**
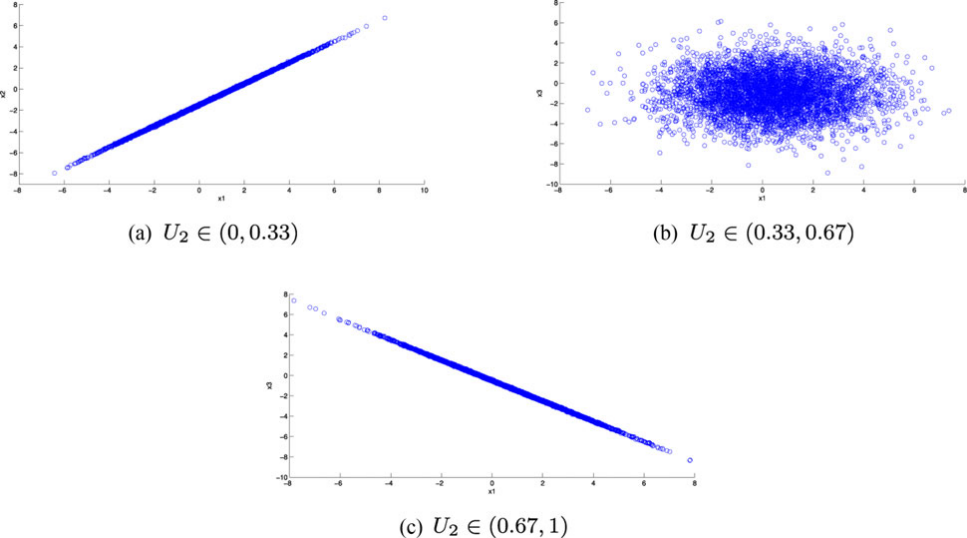
The simulated distributions of $X_{1},X_{3}$ given *X*_2_ in each of the intervals.

We can simulate from the vine to check that we recover the conditional correlations for $X_{1},X_{3}|X_{2}$. In order to do this, we randomly draw $u_{1},u_{2},u_{3}$, three standard uniform variables. Then

$$\begin{array}{c} x_{1}=F_{1}^{-1}\left(u_{1}\right),x_{2}=F_{2|1}^{-1}\left(u_{2}|x_{1}\right),x_{3}=F_{3|12}^{-1}\left(u_{3}|x_{1},x_{2}\right), \end{array}$$

where the distribution function $F_{3|12}$ is found from $f_{3|1,2}\left(x_{3}|x_{1},x_{2}\right)$. For further details on this, see Ref. 15 and Section 5.2.1.. We perform 5,000 simulations. The resulting $X_{1},X_{3}$ values are plotted in Fig. 2.

We have recovered the correlations well in each interval. The simulated correlations are $\rho_{X_{1},X_{3}|U_{2}\in \left(0,0.33\right)}=0.9998,$ $\rho_{X_{1},X_{3}|U_{2}\in \left(0.33,0.67\right)}=-0.0032$, and $\rho_{X_{1},X_{3}|U_{2}\in \left(0.67,1\right)}=-0.9998$.

We shall use this approach when considering the example of Aas *et al*.^11^ in Section 5.1.. We believe that incorporating such nonconstant conditional correlations as we lay out in this article would be a useful addition to the R package VineCopula. By using a smooth function for the dependence instead of a piece‐wise constant function as in the binning in the example above it, would be possible to apply such an approach to large, complex distributions.

The use of Gaussian copulas in financial modeling has come under fire for its uncritical use. Shreve^24^ points out that the simple modeling of correlation available in the Gaussian copula does not pass validation tests, but that this did not stop its widespread adoption in the finance community.

## 3. BUILDING BIVARIATE MINIMUM INFORMATION COPULAS

The emphasis in this article is on approximation rather than on statistically optimal estimation techniques. We use minimum information methods to operationalize the approximation in the class of copulas used. This section discusses how the data required to specify bivariate copulas can be derived, either from expert or sampling data, and shows how this can be used to determine a minimum information copula.

We recall that when *f* and *g* are bivariate densities then the *relative information* of *f* with respect to *g* is:

I(f|g)=∫∫ln(f(x,y)/g(x,y))f(x,y)dxdy.

Information is a measure of the degree of deviation of *f* from *g* and is minimized at 0 when f=g. Furthermore, because the information function is transformation‐invariant, the relative information of *f* with respect to *g* is the same as that between the copula for *f* with respect to the copula of *g*. This makes information a natural quantity with which to measure the degree of dependency in a copula, for if *g* is an independent bivariate with the same marginal distributions as *f*, then I(f|g) is the same as the information of the copula of *f* relative to the independent copula.

From the perspective of information and entropy,^25^ a natural way to specify dependency constraints is through the use of moments. These can be specified either on the copula or on the underlying bivariate density (as long as we know the marginal distributions and can therefore transform from one to the other). We consider moment constraints in which real‐valued functions $h_{1},...,h_{k}$ are required to take expected values $\alpha_{1},...,\alpha_{k}$. By a minimum information copula, we mean a copula that satisfies a set of constraints as above and that has minimum information (with respect to the uniform copula c(u,v)=uv) among the class of all copulas satisfying those constraints. This copula (when it exists and is unique—which is normally the case) is the “most independent” bivariate density that meets the constraints. Note that probabilities are simply expectations of identity functions and so this method of specifying constraints is not restrictive.

Information has a further advantage for us in that it is a natural measure for vine distributions: a specification of minimum information bivariate copulas automatically gives minimum information vine distributions. Specifically, lemma 4.4 and theorem 4.5 in Bedford and Cooke^13^ (see also Kurowicka and Cooke^14^) show that if we take a minimal information copula satisfying each of the (local) constraints (on moments, rank correlation, etc.), then the resulting joint distribution is also minimally informative given those constraints.

### 3.1. Data: Expert Judgment or Random‐Sample‐wBased Approaches

Quantitative operations research models are typically quantified either by expert judgment or estimation from data. In our case, the minimum information models are parameterized by the expected values of functions $h_{i}:{\left[0,1\right]}^{2}\to R$ discussed above. The simplest case is to consider a single function defined on the copula parameters h(u,v)=uv. Specifying the expected value of this is equivalent to specifying the Spearman rank correlation coefficient for the copula.^26^ If we wanted to consider the product‐moment correlation, this would entail transforming back to the original variables and using the function:

$$h\left(u,v\right)=F_{1}^{-1}\left(u\right)F_{2}^{-1}\left(v\right),$$

where *F*_1_ and *F*_2_ are the marginal distributions of the original variables. The use of experts to specify correlations has been explored extensively in the literature (see, for example, Clemen and Reilly^7^). Hence, the methods we propose allow for common correlation‐based approaches to specifying dependence, as well as providing for a wider range of constraints if desired. Kurowicka *et al*.^27^ explored the use of Bayesian networks to structure the specification of parameters for vine models.

We remark that the Spearman correlation can take any value between −1 and +1, whereas the product‐moment correlation is typically restricted to a narrower interval depending on the marginal distributions involved. Bedford^28^ discussed the possibilities of using the minimum information approach to explore the range of feasible values to aid experts in choosing consistent parameter values.

The approach taken in this context is subjectivist and follows a tradition in which expectation values are used to specify uncertain quantities.^25^, ^29^, ^30^, ^31^ Within a conventional Bayesian approach, our work may be thought of as a way to generate an informative prior distribution. We are not suggesting that the approach be used as an alternative to Bayesian updating. We remark that MCMC methods have been used in conjunction with vines^16^, ^32^ in order to update vines.

The elicitation of a joint probability distribution from experts or the approximation of a joint distribution of multiple uncertain quantities are among the key research areas in risk assessment, and the distinction between sources of uncertainty often comes into play in the elicitation of the uncertain quantities.^33^, ^34^ Uncertainties are sometimes distinguished as being either aleatory (stochastic) or epistemic. The former arises because of natural, unpredictable variation in the performance of the system under study. In this case, the proposed method in this article can be used to approximate the joint distribution based on observed sample data for multiple uncertain quantities. Epistemic uncertainty is due to a lack of knowledge about the behavior of the system. This is conceptually resolvable.

The epistemic uncertainty can, in principle, be eliminated with sufficient study. Borgonovo^35^ and Aven^33^ reported that subjective probabilities are often used for representing this type of uncertainty, but several other approaches can be used to represent this uncertainty. Therefore, our method can be used to elicit the prior distribution of unknown parameters by building a subjective multivariate distribution based on observable quantities. Although one may use rank correlations that are not observable quantities, within a minimum information framework it is possible to specify the expected value of any particular function on the probability space. Rank correlation falls into this framework as it is linearly related to the expected value of a product of cdfs in the copula space.

If we wish to fit distributions on the basis of sampling data (large quantities of which may be available, for example, in financial risk modeling problems), the data can be transformed to uniform after estimation of the marginals. This makes it possible to consider approximation, or encoding, of the data using a multivariate copula, and enables us to consider ways of judging how well that approximation can be made using given families of two‐dimensional copulas. We give examples later in the article to illustrate this approach.

### 3.2. The $D_{1}AD_{2}$ Algorithm and Minimum Information Copulas

Suppose there are *k* functions, $h_{1},h_{2},...,h_{k}:{\left[0,1\right]}^{2}\to R$, for which we specify the mean values $\alpha_{1},...,\alpha_{k}$ that these functions simultaneously take. Further suppose that $h_{i},h_{j}$ are linearly independent for i≠j. We seek a copula that has these mean values, a problem that is usually either infeasible or underdetermined. Assuming feasibility for the moment, we ask that the copula be minimally informative (relative to the uniform distribution), which guarantees a unique and reasonable solution. Define the kernel:

(4) $$A\left(u,v\right)=\exp(\lambda_{1}h_{1}\left(u,v\right)+...+\lambda_{k}h_{k}\left(u,v\right)).$$

According to the general theory of Borwein *et al*.^36^ and Nussbaum^37^ (section 4.), there is a unique copula with minimum information satisfying the constraints that the mean value of $h_{i}$ is $\alpha_{i}$ (i=1,...,k), and this has density

$$d^{\left(1\right)}\left(u\right)d^{\left(2\right)}\left(v\right)A\left(u,v\right)$$

for some functions $d^{\left(1\right)}\left(\cdot \right)$, $d^{\left(2\right)}\left(\cdot \right)$. The parameters $\left(\lambda_{1},...,\lambda_{k}\right)$ depend on $\left(\alpha_{1},...,\alpha_{k}\right)$ in a nonlinear way. There are numerical procedures to determine this relationship: given $\left(\lambda_{1},...,\lambda_{k}\right)$ we can numerically determine the functions $d^{\left(1\right)}\left(u\right)$ and $d^{\left(2\right)}\left(v\right)$ and calculate the associated mean values for $h_{1},h_{2},...,h_{k}$. By numerically solving this function, as discussed below, we can find the unique $\left(\lambda_{1},...,\lambda_{k}\right)$ for which the mean values of $h_{1},h_{2},...,h_{k}$ are $\alpha_{1},...,\alpha_{k}$. A summary of the theory based on Bedford and Meeuwissen,^26^ Nussbaum^37^ (section 4.), and Borwein *et al*.^36^ is addressed in Ref. 38.

The general theory says that the set of all possible expectation vectors $\left(\alpha_{1},...,\alpha_{k}\right)$ that could be taken by $\left(h_{1},h_{2},...,h_{k}\right)$ under some probability distribution is convex, and that for every $\left(\alpha_{1},...,\alpha_{k}\right)$ in the interior of that convex set there is a density with parameters $\left(\lambda_{1},...,\lambda_{k}\right)$ for which $\left(h_{1},h_{2},...,h_{k}\right)$ take these expectations.

This general approach to defining a copula was used by Bedford and Meeuwissen^26^ with a single function h(u,v)=uv, which measures the Spearman rank correlation of the copula. Bedford^28^ and Lewandowski^39^ have considered larger groups of functions.

The discrete version of this problem can be written in terms of matrices. In this case, the probability densities defined above are approximated by probability mass functions, which are given below. Suppose that (u,v) are discretized into *n* points, respectively, as $u_{i}$, and $v_{j}$, i,j=1,...,n. Then, we write $A=\left(a_{ij}\right),D_{1}=diag\left(d_{1}^{\left(1\right)},...,d_{n}^{\left(1\right)}\right)$, $D_{2}=diag\left(d_{1}^{\left(2\right)},...,d_{n}^{\left(2\right)}\right)$, where $a_{ij}=A\left(u_{i},v_{j}\right)$, $d_{i}^{\left(1\right)}=d^{1}\left(u_{i}\right)$, $d_{j}^{\left(2\right)}=d^{2}\left(v_{j}\right)$. The assumption of uniform marginals means that:

$$\begin{array}{ccc} \sum_{j}d_{i}^{\left(1\right)}d_{j}^{\left(2\right)}a_{ij} & = & 1/n,\;\mathrm{and}\;\sum_{i}d_{i}^{\left(1\right)}d_{j}^{\left(2\right)}a_{ij}=1/n,\;\;\\ & & \forall i,j=1,...n. \end{array}$$

Hence,

di(1)=1n∑jdj(2)aij and dj(2)=1n∑idi(1)aij.

Finding matrices *D*_1_ and *D*_2_ so that $D_{1}AD_{2}$ is a stochastic matrix has been long studied. Sinkhorn and Knopp^40^ gave a simple algorithm, and the iterative proportional fitting (IPF) algorithm^41^ has been much used. IPF uses an iterative procedure to determine the entries of *D*_1_ and *D*_2_. The idea is simple: start with arbitrary positive initial matrices for *D*_1_ and *D*_2_, then successively define new vectors by iterating the maps:

$$\begin{array}{ccc} & & d_{i}^{\left(1\right)}\mapsto \frac{1}{n\sum_{j}d_{j}^{\left(2\right)}a_{ij}}\;\left(i=1,...,n\right),\\ & & d_{j}^{\left(2\right)}\mapsto \frac{1}{n\sum_{i}d_{i}^{\left(1\right)}a_{ij}},\;\;\left(j=1,...,n\right). \end{array}$$

This iteration converges geometrically to give us the vectors required. Nussbaum^37^ (section 4.) considered the problem in greater generality, considering continuous densities and functions, and showed that the corresponding functional is a contraction mapping on a space of functions endowed with a Hilbert projective metric. We make use of this fact when considering the quality of approximations made to copulas below.

To compare different discretizations (for different *n*), we multiply each cell weight $d_{i}^{\left(1\right)}d_{j}^{\left(2\right)}a_{ij}$ by *n* ^2^ as this quantity approximates the continuous copula density with respect to the uniform distribution.

As discussed above, for a given set of functions $\left(h_{1},...,h_{k}\right)$, the mapping from the set of vectors of λs parameterizing the kernel *A* onto the expectations of the function $\left(\alpha_{1},...,\alpha_{k}\right)$ is found numerically, and optimization techniques are used. We wish to determine the appropriate set of λs for given expectations $\alpha_{i}$, where the expectations are calculated using the discrete copula density $D_{1}AD_{2}$. Define

(5) $$\begin{array}{ccc} L_{l}\left(\lambda_{1},...,\lambda_{k}\right): & = & \sum_{i=1}^{n}\sum_{j=1}^{n}d^{\left(1\right)}\left(u_{i}\right)d^{\left(2\right)}\left(v_{j}\right)A\left(u_{i},v_{j}\right)\\ & & h_{l}\left(u_{i},v_{j}\right)-\alpha_{l},\;\;\;l=1,2,...,k. \end{array}$$

We seek the simultaneous roots of these functions and so minimize

$$L_{sum}\left(\lambda_{1},...,\lambda_{k}\right)=\sum_{l=1}^{k}L_{l}^{2}\left(\lambda_{1},...,\lambda_{k}\right).$$

The problem can be solved using one of Matlab's optimization procedures, FMINSEARCH, which implements the Nelder‐Mead simplex method.^42^ This is used in the examples in this article.

We remark that, given the choice of functions $\left(h_{1},...,h_{k}\right)$, we have a parametric class of distributions with parameters the expected values $\left(\alpha_{1},...,\alpha_{k}\right)$ of $\left(h_{1},...,h_{k}\right)$. However, although we have a parametric family, we do not have a closed‐form expression for that family. Although the kernel in Equation (4) has a closed‐form expression, the functions *d* ^(1)^ and *d* ^(2)^ do not. They are, however, uniquely defined and simple to compute. Pseudo‐code is given in the Supporting Information to the article.

When fitting common parametric copulas such as the *t*‐copula using expert judgment it can be difficult to relate the parameters of the copula to observable quantities for which we can ask experts values. This is not true using minimum information copulas, however, due to the flexibility of the functions $h_{i}\left(\cdot \right)$. As an example, we show how an expert could specify a copula though defining two expected values.

### 3.3. Example

Suppose *X* and *Y* represent the failure times of two components that are functionally identical and physically colocated. There are many reasons to believe that the distributions of *X* and *Y* will be dependent, but modeling all the different sources of dependency may be difficult to do explicitly. Assume that the marginal distribution functions $F_{X}$ and $F_{Y}$ are exponential with mean time to failure 100, and that we want to specify a copula for (X,Y).

We could ask an expert for information about the likelihood of near‐simultaneous failure. Suppose that the expert assesses that the probability of both systems failing within time 1 of each other is 0.1, and that the probability both systems fail within time 10 of each other is 0.3. The expert information says that if we consider the functions of the copula variables *U* and *V*, defined by:

h1(u,v)=1 if |FX−1(u)−FY−1(v)|<10 otherwise ,,h2(u,v)=1 if |FX−1(u)−FY−1(v)|<100 otherwise ,

then the copula needs to be chosen so that the expected value of *h*_1_ is 0.1 and that of *h*_2_ is 0.3. Using the methods discussed here we can construct the minimum information copula.

In general, the range of expectation values available to the expert will be constrained, in the first instance by the choice of marginal distribution, and then by the expected value chosen for the first function. This was discussed by Bedford^28^ in the context of a single copula. Two important aspects are discussed there. First, we can choose functions for evaluation that have a real “operational meaning” for the experts, which is better than asking them to assess moments or abstract parameter values. Second, as the range of possible values for the expectation of a function can be computed by evaluating the function's expected value as we change the Lagrange multiplier values in Expression (4), we can offer guidance to experts about what values may be chosen to be consistent with those already chosen.

**Figure 3:**
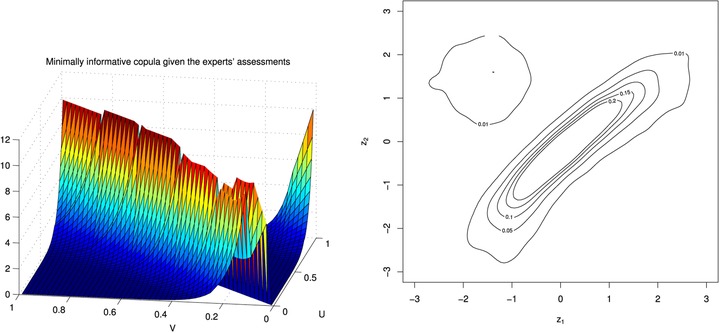
A plot of the minimum information copula and transformed contour plot for X,Y.

The resulting parameter values for this copula, found using a discretized grid of 200 × 200 points, are $\lambda_{1}=-12.9100$ and $\lambda_{2}=-1.377$. The left‐hand side of Fig. 3 shows the minimally informative copula for these values.

On the right‐hand side of Fig. 3, we have included a contour plot of the copula density transformed to allow for standard normal margins by transforming the copula coordinates (u,v) to $\left(z_{1},z_{2}\right)$ with $z_{j}=\Phi^{-1}\left(u_{j}\right)$ for j=1,2. This allows us to assess which is the closest bivariate copula to the minimum information copula fitted and so allows comparison to common parametric copulas. In the case of a Gaussian copula, the contour plots will be elliptical, while shapes like pears give indication of tail dependence induced, for example, by a Clayton or Gumbel copula. Bivariate *t*‐copulas are identified through diamond‐shaped contours. In this case, we see an elliptical shape.

## 4. COPULA COMPACTNESS

The previous section showed how bivariate minimum information copulas can be constructed and provides a useful family of bivariate copulas. However, the article aims to construct higher dimensional copulas. An important technical step is taken in this section where we consider the amount of variability between different bivariate copulas arising in a multivariate copula. The key step is to show that the family of bivariate (conditional) copula densities contained in a given multivariate copula distribution forms a compact set in the space of continuous functions on [0, 1]^2^. We can then show that the same finite parameter family of copulas can be used to give a given level of approximation to all conditional copulas simultaneously.

It is important to define precisely the way in which we approximate densities. We assume that all densities are continuous and uniformly bounded away from zero. Write C(Z) for the space of continuous real‐valued functions on a space *Z*, where we shall always take $Z={\left[0,1\right]}^{r}$ for some *r*. A norm on the space C(Z) is given by:

$$\left|\right|f_{1...r}\left||=\sup\right|f_{1...r}\left(x_{1},...,x_{r}\right)|,\;f_{1...r}\in C\left(Z\right).$$

Since our functions are assumed continuous on *Z*, and since *Z* is compact, the norm of any such function is finite. We shall be interested in the set of all possible two‐dimensional (conditional) copulas associated to a given continuous density function *f*:

$$\begin{array}{ccc} C(f) & = & \{c_{ij|i_{1}...i_{r}}:1\leq i,j,i_{1},...,\\ & & i_{r}\leq n,i,j\neq i_{1},...,i_{r}\}, \end{array}$$

where $c_{ij|i_{1}...i_{r}}$ is the copula of the conditional density of $X_{i},X_{j}$ given $X_{i_{1}},...,X_{i_{r}}$. Thus, C(·) is an infinite set. It will be important to show that this set is relatively compact in the space of all continuous real‐valued functions *C*([0, 1]^2^) because then we can show that the copula densities can be uniformly approximated. We consider compactness relative to the topology induced by the sup norm.

Compactness of a set *K* can be defined equivalently through one of two properties, each of which we shall use. (1) Any open cover of *K* has a finite subcover. In other words, if *K* is a subset of an infinite union of open sets, then it is in fact also a subset of a finite union of those open sets. (2) Any sequence of points (which in our case are functions) of *K* has a convergent subsequence.

The Arzela‐Ascoli Theorem gives another way of checking compactness when dealing with function spaces. It says that a subset $K\subset C({\left[0,1\right]}^{2})$ is relatively compact if the functions of *K* are equicontinuous and point‐wise bounded. We recall that a set of functions is equicontinuous if for all ε>0 and (u,v) there is a δ>0 such that if the Euclidean distance $|\left(u,v\right)-\left(u^{\prime },v^{\prime }\right)|<\delta$ then $|g\left(u,v\right)-g\left(u^{\prime },v^{\prime }\right)|<\varepsilon \forall g\in K,$ and that *K* is pointwise bounded if sup{||g||:g∈K}<∞.

As a first step to showing the relative compactness of C(f), we first consider two other spaces: the set of conditional marginal densities:

$$\begin{array}{c} M\left(f\right)\;=\;\left\{f_{i|i_{1}...i_{r}}:1\;\leq \;i,i_{1},...,i_{r}\leq n,i\;\neq \;i_{1},...,i_{r}\right\}, \end{array}$$

where $f_{i|i_{1}...i_{r}}\left(x_{i}|x_{i_{1}},...,x_{i_{r}}\right):\left[0,1\right]\to R$ are the conditional densities of $X_{i}$ given $X_{i_{1}},...,X_{i_{r}}$, one function for each combination of conditioning values $x_{i_{1}},...,x_{i_{r}}$, and the set of conditional bivariate densities:

$$\begin{array}{ccc} B(f) & = & \{f_{ij|i_{1}...i_{r}}:1\leq i,j,i_{1},...,i_{r}\leq n,i,\\ & & j\neq i_{1},...,i_{r}\}, \end{array}$$

where $f_{ij|i_{1}...i_{r}}$ is the conditional density of $X_{i},X_{j}$ given $X_{i_{1}},...,X_{i_{r}}$. Thus, M(f),B(f) are also infinite sets. As we have defined it, a member of M(f) is a function of one variable—in other words, all the different marginals that we get for different conditions are individually members of M(f). Similarly for B(f). Hence, M(f)⊂C([0,1]) and $B\left(f\right)\subset C({\left[0,1\right]}^{2})$.

THEOREM 2 The sets M(f)⊂C([0,1]) and $B\left(f\right)\subset C({\left[0,1\right]}^{2})$ are relatively compact.

See Appendix A.□

THEOREM 3 The set $C\left(f\right)\subset C({\left[0,1\right]}^{2})$ is relatively compact.

See Appendix A.□

Since all the functions in C(f) are positive and uniformly bounded away from 0 it follows that:

COROLLARY 1 The set $\mathrm{LNC}\left(f\right)=\left\{\ln\left(g\right):g\in C\left(f\right)\right\}\subset C\left({\left[0,1\right]}^{2}\right)$ is relatively compact.

### 4.1. Linear Bases and Approximate Copulas

Consider the ordered set of sequences $h_{0},h_{1},h_{2},...\subset C\left({\left[0,1\right]}^{2}\right)$. We would like any finite sequence $h_{0},h_{1},...,h_{n}$ to be linearly independent modulo the constants. The set *C*([0, 1]^2^) can be considered a vector space. Define $V_{n}$ to be the vector space generated by the first *n* terms in the sequence. We would also like to show that $\cup_{n}V_{n}$ is dense in *C*([0, 1]^2^).

A countable basis $h_{0},h_{1},...$ of *C*([0, 1]^2^) over the field R is a countable subset $h_{0},h_{1},...\subset C\left({\left[0,1\right]}^{2}\right)$ with the property that every element $v\in C({\left[0,1\right]}^{2})$ can be written as an infinite series $v=\sum_{i=0}^{\infty }\lambda_{i}h_{i},$ in exactly one way, where $\lambda_{i}\in R$.

Consider the countable basis $h_{0},h_{1},...$. Since v=0 can be written in exactly one way, then this must be with $\lambda_{i}=0$ for all *i*. This means that any finite collection of basis elements is linearly independent modulo the constants. If we set $h_{0}=1$, then, for any *n*, $h_{1},...,h_{n}$ are linearly independent. It is also clear that $\cup_{n}V_{n}$ is dense in *C*([0, 1]^2^). There are lots of possible bases, for example, $u,v,uv,u^{2},v^{2},u^{2}v,uv^{2},....$

Given an ordered basis $h_{1},h_{2},...\in C\left({\left[0,1\right]}^{2}\right)$ and a required degree of approximation ε>0 in the sup metric, we can consider the collection of open sets:

$$U_{k,\varepsilon }=\{g\in C\left({\left[0,1\right]}^{2}\right):\inf||g-\sum_{i=1}^{k}\lambda_{i}h_{i}||<\varepsilon \},$$

where the infimum in the above definition is to be taken over all possible values of the $\lambda_{i}$. Now, $U_{k,\varepsilon }$ is clearly open and furthermore:

$$U_{k,\varepsilon }\subset U_{k+1,\varepsilon },\;\overset{\infty }{\underset{k=1}{⋃}}U_{k,\varepsilon }=C\left({\left[0,1\right]}^{2}\right).$$

So the $U_{k,\varepsilon }$ form an open cover of LNC(f) and hence by definition of compactness there is a *k* such that $U_{k,\varepsilon }$ covers LNC(f). We can state this as a result.

THEOREM 4 Given ε>0, there is a *k* such that any member of LNC(f) can be approximated to within error ε>0 by a linear combination of $h_{1},h_{2},...,h_{k}$.

The same result holds for C(f) (though not necessarily with the same *k*). We call the linear combination $\sum_{i=1}^{k}\lambda_{i}h_{i}$ an approximate copula because it is not guaranteed to be a copula itself. The next section shows that it can be adjusted slightly to obtain a copula that provides good approximation.

We remark that though we have been looking at approximation in the sense of the sup norm, one could easily look at higher order approximation. For example, if we assume that the density $f_{1...n}$ is continuously differentiable, then all the derivatives are continuous functions and the same arguments as used above show that they form an equicontinuous and point‐wise bounded family. Following through we find that the copulas generated from $f_{1...n}$ are also continuously differentiable. By using a slightly different norm on the continuously differentiable functions $C^{1}\left({\left[0,1\right]}^{2}\right)\subset C\left({\left[0,1\right]}^{2}\right)$,

$${\left||g|\right|}_{1}=\left||g|\right|+\left|\right|\frac{d}{du}g||+\left|\right|\frac{d}{dv}g\left|\right|,$$

we can guarantee that a similar approximation result to the above holds with point‐wise approximation of the derivatives.

### 4.2. Ensuring that Approximating Densities are Copula Densities

Since the approximation we make of a copula density is not guaranteed to be a copula density itself, we need to transform it to obtain a copula. This is done by weighting the density as described in Section 3.2.. If we have a continuous positive real‐valued function A(u,v) on [0, 1]^2^, then there are continuous positive functions $d^{\left(1\right)}\left(u\right)$ and $d^{\left(2\right)}\left(v\right)$ such that $d^{\left(1\right)}\left(u\right)d^{\left(2\right)}\left(v\right)A\left(u,v\right)$ is a copula density, that is, it has uniform marginals. We call this density the *C*‐*Projection* of *A* and denote it C(A). It will be convenient to denote by N(h) the normalization of a nonnegative function *h* with finite integral.

We can control the error made when approximating a copula by another function.

Lemma 1 Let *g* be a nonnegative continuous copula density. Given ε>0 there is a δ such that if ||g−f||<δ then ||g−C(f)||<ε.

See Appendix A.□

The reweighting functions have the same differentiability properties as the function *f* being reweighted. This can be seen from the integral equation that they satisfy:

d(1)(u)=1∫d(2)(v)f(u,v)dv and d(2)(v)=1∫d(1)(u)f(u,v)du.

We use Equation (2) to see that good approximation of each conditional copula gives a good approximation of the multivariate density.

## 5. CONSTRUCTING APPROXIMATIONS USING MINIMALLY INFORMATIVE DISTRIBUTIONS

The above discussion has shown that we can approximate all conditional copulas using linear combinations of basis functions. We did not address the question of how you choose the appropriate parameter values, and finding the parameters that would minimize the sup norm for a given copula is not an appealing procedure. An alternative that lies very close to the approach described above is to use the minimum information criterion. Given $\left\{1,h_{1},...,h_{k}\right\}:{\left[0,1\right]}^{2}\to R$ we seek values $\lambda_{1},...,\lambda_{k}$ so that $\exp(\sum_{1}^{k}\lambda_{i}h_{i})$ is close to the copula density we are approximating.

In the minimum information framework, we do this by fitting the moments of $h_{i}$. So if $\int \int h_{i}gdudv=\alpha_{i}$ then we search for the copula density with minimum information (with respect to the independent distribution) that has those moments. This copula density is unique and has the form:

$$d^{1}\left(u\right)d^{2}\left(v\right)\exp\left(\sum_{i=1}=1^{k}\lambda_{i}h_{i}\left(u,v\right)\right).$$

When we use a vine structure to model a multivariate distribution, the vine defines a decomposition of the multivariate distribution into conditional copulas, associated to the conditioned and conditioning sets of the vine. For example, if {i,j} is the conditioned set and $D_{e}$ is the conditioning set in one part of a vine, then the family of conditional copulas for $x_{i},x_{j}$ given $D_{e}$ has to be specified. Using the minimum information approach means that we specify mean values for the functions $h_{r}$ given the variables in $D_{e}$, that is, we specify the conditional means $\alpha_{m}\left(ij|D_{e}\right)$.

A multivariate distribution can be approximated as follows. Specify a basis family B(k), specify a vine structure and for each part of vine, specify either expected values $\alpha_{1},...,\alpha_{k}$ for $h_{1},...,h_{k}$ on each pairwise copula or functions $\alpha_{m}\left(ji|D_{e}\right)$ for the expected values as functions of the conditioning variables, for m=1,...,k.

We remark that, since under our assumptions there is a uniform lower bound on the density of the copulas used in the representation, the uniform point‐wise approximation that can be achieved implies information convergence in two ways. By making the copula approximations close enough, we can ensure that (i) the information of the overall approximate multivariate copula (with respect to the independent copula) is close to that of the original multivariate copula, and (ii) the information of our approximate multivariate copula relative to the original multivariate copula is close to zero.

We illustrate the procedure by applying it to two financial data sets.

### 5.1. Example: Stock Market Time Series

We use the same data set as considered by Aas *et al*.^11^ We have four time series of daily data: the Norwegian stock index (TOTX), the MSCI world stock index, the Norwegian bond index (BRIX), and the SSBWG hedged bond index. All are for the period January 1, 1999 to July 8, 2003. We denote these four variables T,B,M, and *S*.

We generate a vine approximation fitted to this data set using minimum information distributions. We adopt a vine structure similar to that in Fig. 1 with variables T,B,M,S being 1, 2, 3, 4, respectively. We can find the corresponding functions of the copula variables X,Y,Z, and *W* associated with T,M,B,S. These are defined by, for example, $h_{i}\left(X,Y\right)=h_{i}^{{}^{\prime }}\left(F_{1}^{-1}\left(T\right),F_{2}^{-1}\left(M\right)\right)$ and have the same specified expectation, in this case $E\left[h_{i}^{{}^{\prime }}\left(T,M\right)\right]=E\left[h_{i}\left(X,Y\right)\right]$. The minimum information copulas calculated are derived based on the copula variables X,Y,Z,W.

Initially, we construct minimally informative copulas between each set of two adjacent variables in the first tree, *T*_1_. We must decide on which bases to take and how many discretization points to use in each case. We illustrate the recommended procedure for the first copula in *T*_1_, between T,M.

#### 5.1.1. Step‐wise Inclusion of Basis Functions

**Figure 4:**
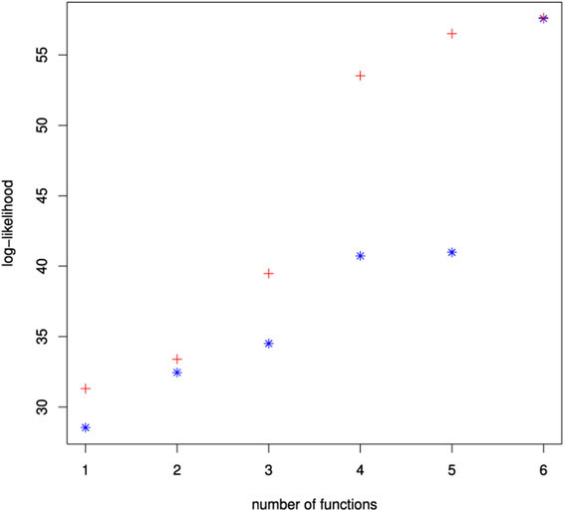
The log‐likelihood of the minimally informative copula calculated based on different functions for the simple (blue stars) and stepwise (red crosses) methods (colors visible in on‐line version).

We wish to know which basis functions to include in our copula. We could choose basis functions, starting with simple polynomials and moving to more complex ones, and include them until we are satisfied with our approximation. For example, if we included the following basis functions in order $TM,TM^{2},T^{2}M,TM^{3},$ $T^{3}M,T^{2}M^{3}$, then the log‐likelihood for the copula changes as in the blue stars in Fig. 4.

There is a jump in the log‐likelihood as we add the sixth basis function, $T^{2}M^{3}$. This could imply that we are not adding the basis functions in an optimal manner. Instead, at each stage, we propose to assess the log‐likelihood of adding each additional basis function. We include the function that produces the largest increase in the log‐likelihood. Our method is similar to a step‐wise regression. Doing so for the initial copula yields the basis functions $TM^{2},T^{2}M,T^{2}M^{2},TM,TM^{4},T^{2}M^{4}$. The log‐likelihood at each stage is given in the red crosses in Fig. 4.

We see that there is no longer a jump in the log‐likelihood when adding the sixth basis function. The log‐likelihood also increases more quickly and reaches its plateau value of around 60 using fewer basis functions. We use this step‐wise technique to choose all of the remaining basis functions in the example. The use of log‐likelihood in this way is not inconsistent with minimum information modeling. Jaynes^25^ uses the parameter maximum likelihood estimates associated with the form of the minimum information distribution to justify the connection in the constraint rule of expectations and frequencies.

#### 5.1.2. Returning to the Example

We include the six basis functions given above, that is, $h_{1}^{\prime }\left(T,M\right)={TM}^{2}$, $h_{2}^{\prime }\left(T,M\right)=T^{2M}$, $h_{3}^{{}^{\prime }}\left(T,M\right)=T^{2}M^{2}$, $h_{4}^{\prime }\left(T,M\right)=TM$, $h_{5}^{\prime }\left(T,M\right)={TM}^{4}$, and $h_{6}^{\prime }\left(T,M\right)=T^{2}M^{4}$. We shall fix the values of the expectations of these functions by using the empirical data. For example, $\alpha_{1}=\frac{1}{1,094}\sum_{i=1}^{1,094}t_{i}m_{i}^{2}$, as there are 1,094 observations for each variable.

The minimum information copula $C_{TM}$ with respect to the uniform distribution given the six constraints above can be constructed. To do so, we need to decide on the number of discretization points (or grid size). A larger grid size will provide a better approximation to the continuous copula but at the cost of more computation time. Similarly, the more iterations of the $D_{1}AD_{2}$ and optimization algorithms that are run, the more accurate the approximation will become. This is again at the expense of speed. Comments on the $D_{1}AD_{2}$ algorithm are given in Section 3.2.. In terms of the optimization, we can specify how accurate we wish our approximation to be and then judge the effect on the number of iterations required for convergence. The number of iterations needed will also depend on the grid size.

**Figure 5:**
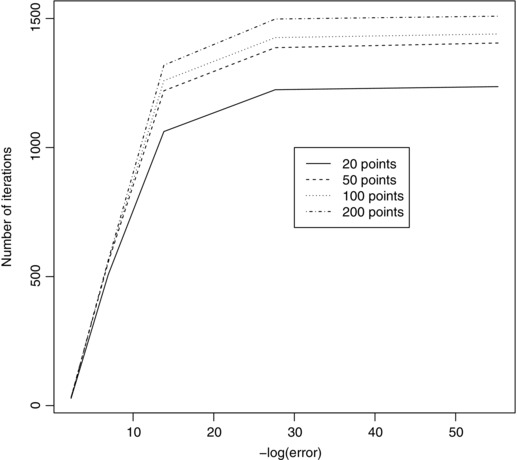
A plot of the number of iterations against convergence level for 20, 50, 100, and 200 discretization points.

Fig. 5 gives a plot comparing the number of iterations required for convergence of FMINSEARCH given a certain error of $L_{sum}$ and grid size. The errors considered are in the range $1\times 10^{-1}$ to $1\times 10^{-24}$. These are then transformed by taking −log(.) and this is the quantity plotted.

We see that the larger the number of grid points used, the larger the number of iterations needed for convergence. This is true over all error levels. The grid sizes all follow the same pattern, with large increases in the number of iterations needed for improved accuracy initially and smaller increases when the error is smaller.

Throughout the rest of the example, we choose a grid size of 200 × 200 and shall work to an error of $1\times 10^{-12}$. This corresponds to a transformed error in Fig. 5 of 27.63. This represents a suitable balance between providing an accurate approximation to the minimally informative copula and keeping computational effort to a reasonable level.

**Figure 6:**
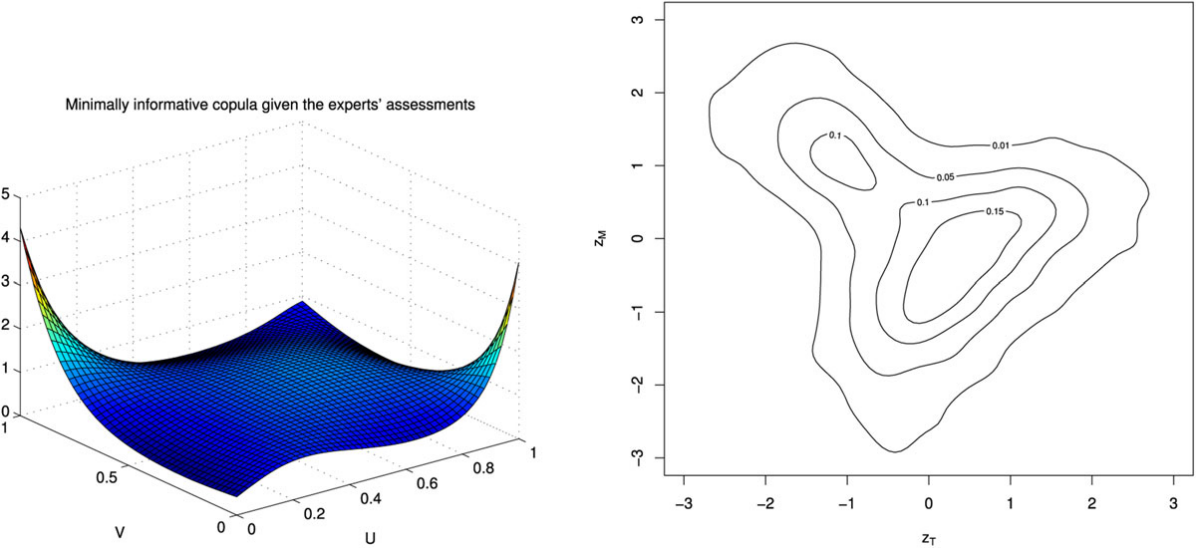
The minimally informative copula between *T* and *M* and transformed contour plot, Norwegian stock data.

We can find the minimally informative copula $C_{TM}$. Pseudo‐code for doing this is given in the Supporting Information. This gives parameter values of $\lambda_{1}=17.0262,\lambda_{2}=-17.6367,\lambda_{3}=-1.1117,\lambda_{4}=4.7746,\lambda_{5}=-26.8054,\lambda_{6}=19.9014.$ The copula is plotted on the left‐hand side of Fig. 6 and the contour plot of the copula density transformed to allow for standard normal margins is given on the right‐hand side. The log‐likelihood for the copula is $l_{\mathrm{TM}}=58.1256$.

The remaining copulas in *T*_1_ are $C_{MB},C_{BS}$. The constraint functions, constraints, and Lagrange multipliers used for this copula are given in Table I. The log‐likelihoods are $l_{\mathrm{MB}}=155.18$ and $l_{\mathrm{BS}}=19.23$, respectively.

**Table I:** Constraints and Parameter Values for $C_{MB}$ and $C_{BS}$

$h_{i}^{{}^{\prime }}\left(M,B\right)$	$\alpha_{i}$	$\lambda_{i}$	$h_{i}^{{}^{\prime }}\left(B,S\right)$	$\alpha_{i}$	$\lambda_{i}$
MB	0.2905	24.970	BS	0.2375	18.818
$M^{2}B$	0.2066	−22.233	$B^{2}S$	0.1546	−26.914
$M^{3}B$	0.1611	20.308	$B^{3}S$	0.1142	7.929
$M^{2}B^{3}$	0.1223	32.006	$B^{3}S^{2}$	0.0730	−13.949
$M^{2}B^{2}$	0.1527	−39.639	$BS^{2}$	0.1537	−24.939
$MB^{5}$	0.1142	−3.910	$B^{2}S^{2}$	0.0992	36.763

The conditional copulas in the second tree, *T*_2_, can be approximated using the minimum information approach. Initially, we construct the conditional minimum information copula between T∣M and B∣M. Aas *et al*.^11^ considered the dependencies between *T* and *B* given *M* to be constant over M∈[0,1].

Instead, we divide the support of *M* into some arbitrary subintervals or bins and then construct the conditional copula within each bin. We will investigate the effect of this in the following example. We find bases in the same way as for the marginal copulas and fit the copulas to the expectations calculated for these. We use four bins so that the first copula is for T,B∣M∈(0,0.25). The bases for this copula are $h_{1}^{{}^{\prime }}\left(T,B|M\in \left(0,0.25\right)\right)=T^{2}B,h_{2}^{{}^{\prime }}\left(T,B|M\in \left(0,0.25\right)\right)=T^{3}B,h_{3}^{{}^{\prime }}\left(T,B|M\in \left(0,0.25\right)\right)=T^{4}B,h_{4}^{{}^{\prime }}\left(T,B|M\in \left(0,0.25\right)\right)=T^{5}B,h_{5}^{{}^{\prime }}\left(T,B|M\in \left(0,0.25\right)\right)=TB^{5},h_{6}^{{}^{\prime }}\left(T,B|M\in \left(0,0.25\right)\right)=T^{2}B^{4}.$ The expectations given these basis functions that will constrain the minimum information copula are $\alpha_{1}=0.1246,\alpha_{2}=0.0983,\alpha_{3}=0.0813,\alpha_{4}=0.0693,\alpha_{5}=0.0239$, and $\alpha_{6}=0.0220$.

We follow this process again for the remaining bins. Table II shows the constraints and corresponding Lagrange multipliers required to build the conditional minimum information copula between T∣M∈(0,1) and B∣M∈(0,1). The overall log‐likelihood of the conditional minimum information copula between *T* and *B* given M∈(0,1) is 29.242.

**Table II:** Bases, Parameter Values, and Log‐Likelihoods for $C_{TB|M}$

Interval	Bases	Parameter Values
0<M<0.25	$\left(T^{2}B,T^{3}B,T^{4}B,T^{5}B,TB^{5},T^{2}B^{4}\right)$	(26.0,−141.5,231.8,−120.0,12.4,10.6)
0.25<M<0.5	$\left(TB,TB^{2},T^{3}B,T^{4}B,T^{2}B,T^{2}B^{3}\right)$	(−32.4,16.0,−188.2,112.2,103.3,−9.2)
0.5<M<0.75	$\left(T^{2}B,TB^{2},T^{3}B,T^{2}B^{3},TB,TB^{5}\right)$	(13.4,33.6,12.1,−22.2,−35.0,−4.2)
0.75<M<1	$\left(TB^{2},TB^{3},TB^{4},TB,T^{5}B,TB^{2}\right)$	(−22.5,38.5,−23.6,1.7,−3.6,6.7)

Similarly, we can construct the minimum information copula between M∣B and S∣B based on four bins and six constraints. The resulting minimum information copula has a log‐likelihood of 16.3901.

The conditionally minimally informative copula in the third tree, *T*_3_, can be obtained. We first divide each of the conditioning variables' supports into four bins as in *T*_2_. Then, the minimum information copulas for T∣(M,B) and S∣(M,B) are calculated on each combination of bins for M,B. In *T*_3_, there are 16 bins altogether. Details are omitted. The log‐likelihood of *T*_3_ is 110.69.

The log‐likelihood of the overall vine, obtained by summing the log‐likelihoods of each of the component copulas, is 388.859. This is larger than that using the vine construction of bivariate *t*‐copulas and constant conditional dependence of Aas *et al*.^11^ of 291.801. Suppose, rather than choosing our bases using the step‐wise method, we had calculated all of the copulas using the same six basis functions. Further suppose that those chosen were the simple polynomials, $XY,XY^{2},X^{2}Y,XY^{3},X^{3}Y,X^{2}Y^{3}$. Then the overall log‐likelihood is 370.147. This is lower than when using our approach but better than the *t*‐copula of Aas *et al*. However, the advantage of the step‐wise method can be seen if we take fewer basis functions for each copula. If we take 5, we obtain a log‐likelihood of 377.552, which is still larger than that obtained using 6 without the step‐wise approach.

### 5.2. Example: Comparison with the Gaussian Copula

We consider five years of exchange rates against the U.S. dollar for four different currencies: the Great British Pound, the Euro, the Japanese Yen, and the South Korean Won. Before fitting the copula models to the data, we first remove any trends, seasonality, etc., from the data by fitting ARMA(p,q)−GARCH(r,s) models to each of the individual time series. The analysis is then conducted on the empirical cdf values of the residuals from the time‐series models. For more details on this, see Ref. 11.

In order to fit a four‐dimensional D‐vine to the data, we need to identify a structure for the vine. Using the methods in this article, we know that we can fit any vine structure arbitrarily well using bivariate minimum information copulas. However, we select the structure using the method given in the VineCopula package in R. This identifies the structure of the vine sequentially, modeling the strongest correlations in the first tree of the vine, assuming that the bivariate copulas do not change with the conditioning value. Further information is given in Ref. 19.

The resulting structure of the D‐vine gives Euros, Great British Pounds, South Korean Won, and Japanese Yen, respectively, in the first tree. We relabel these currencies 1, 2, 3, and 4.

The Gaussian copula has been criticized for its widespread use in the financial sector in spite of evidence that the assumptions underlying modeling and necessary for use were not being met.^43^ One such assumption of the Gaussian copula is that the conditional dependencies between variables in the model are constant. We apply the Gaussian copula, as well as a minimum information vine structure, to the exchange rate data to investigate the suitability of this assumption.

The four‐dimensional Gaussian copula for the currencies takes the form:

$$\begin{array}{ccc} C_{\Sigma }\left(x_{1},x_{2},x_{3},x_{4}\right) & = & \Phi_{\Sigma }\left(\Phi^{-1}\left(F_{1}\left(x_{1}\right)\right),\Phi^{-1}\left(F_{2}\left(x_{2}\right)\right),\right.\\ & & \left.\Phi^{-1}\left(F_{3}\left(x_{3}\right)\right),\Phi^{-1}\left(F_{4}\left(x_{4}\right)\right)\right), \end{array}$$

where $\Phi_{\Sigma }\left(\cdot ,\cdot ,\cdot ,\cdot \right)$ is the cdf of the 4‐variate standard Gaussian distribution with mean zero and variance matrix Σ, $\Phi^{-1}\left(\cdot \right)$ represents the inverse cdf of the univariate standard Gaussian distribution, and $F_{i}\left(\cdot \right)$ represents the cdf for currency i=1,2,3,4.

We first fit a Gaussian copula to the residual series. The fitted values for the correlations are:

(6) $$\begin{array}{ccc} \rho_{12} & = & 0.61,\rho_{13}=0.31,\rho_{14}=-0.058,\rho_{23}=0.35,\\ \rho_{24} & = & 0.027,\rho_{34}=-0.081. \end{array}$$

We fit a minimum information vine and compare the two approaches by simulating from the two distributions. We fit a D‐vine in four dimensions. This requires a minimum information copula specified between exchange rates 1 and 2, one between 2 and 3, and another between rates 3 and 4, a conditional copula between rates 1 and 3 given exchange rate 2 and between rates 2 and 4 given exchange rate 3, and a conditional copula between rates 1 and 4 given exchange rates 2 and 3. We specify four basis functions for each copula and use the same basis functions each time, namely, $h_{1}\left(u_{i},u_{j}\right)=u_{i}u_{j},h_{2}\left(u_{i},u_{j}\right)=u_{i}^{2}u_{j},h_{3}\left(u_{i},u_{j}\right)=u_{i}u_{j}^{2},h_{4}\left(u_{i},u_{j}\right)=u_{i}^{2}u_{j}^{2}$ for i=1,2,3,j≠i. We could have used the method from the previous example to choose the optimal basis functions.

**Figure 7:**
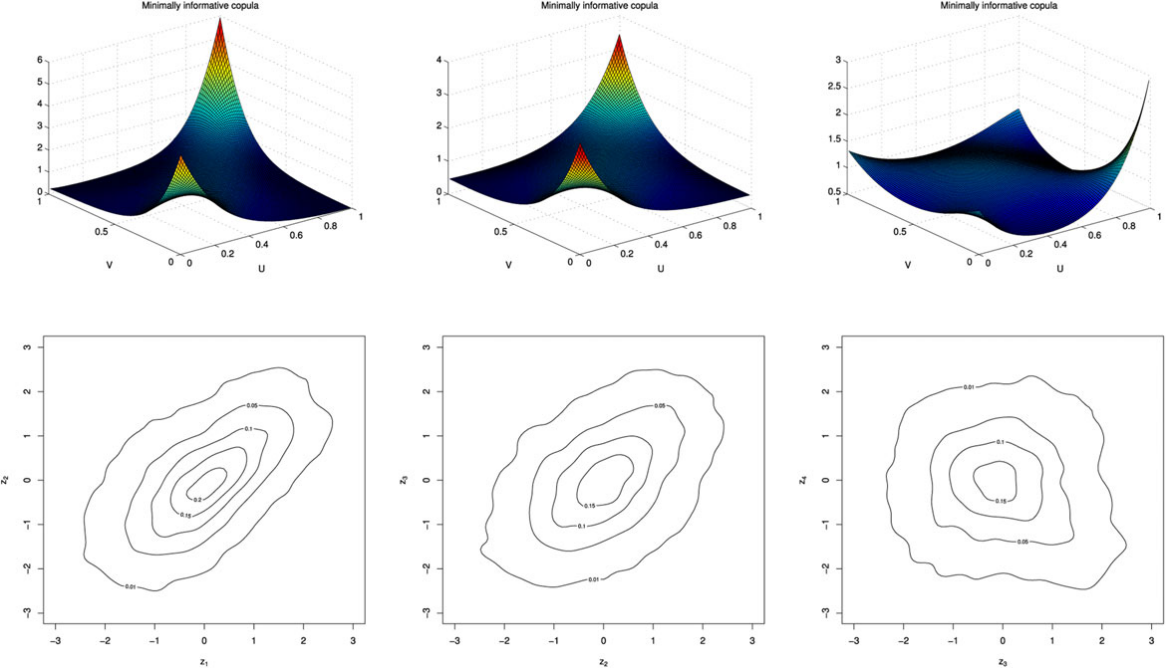
The bivariate minimum information copulas (top) and transformed contour plots (bottom) for the exchange rates of currencies 1 and 2, 2 and 3, and 3 and 4, respectively.

Table III gives a summary of the constraints and resulting parameter values for the marginal copulas. The copulas are given in the top three plots of Fig. 7 and the contour plot of the copula density transformed to allow for standard normal margins are given, respectively, at the bottom of the figure.

**Table III:** The Constraints and Lagrange Multipliers for the Three Marginal Copulas in the First Tree of the Vine

Copula		
Variables	$\left(\alpha_{1},\alpha_{2},\alpha_{3},\alpha_{4}\right)$	$\left(\lambda_{1},\lambda_{2},\lambda_{3},\lambda_{4}\right)$
$u_{1},u_{2}$	(0.301,0.218,0.219,0.166)	(33.63,−20.15,−33.90,30.17)
$u_{2},u_{3}$	(0.280,0.196,0.197,0.142)	(26.22,−21.69,−22.49,22.21)
$u_{3},u_{4}$	(0.244,0.162,0.159,0.105)	(21.36,−25.22,−18.89,21.88)

We wish to split the support of *u*_2_ into bins and define the conditional copula for *u*_1_ and *u*_3_ based on these bins. After plotting the conditional correlations for several different numbers of bins, we settle on four bins.

**Figure 8:**
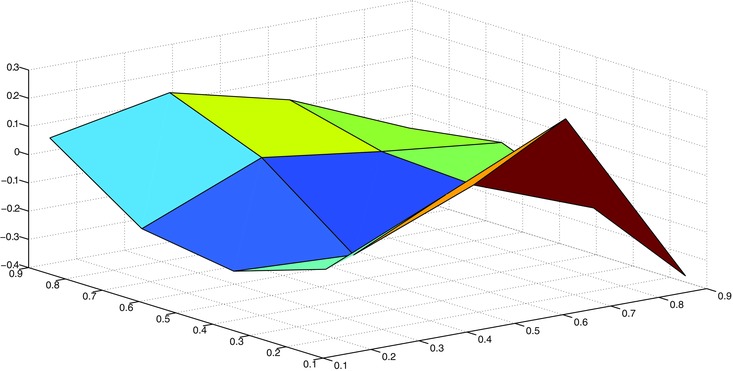
The changes in conditional correlation between exchange rates 1 and 4 given different bins for exchange rates 2 and 3.

The remaining copula is that between *u*_1_ and *u*_4_. To construct this, we must create bins of combinations of *u*_2_ and *u*_3_. We separate *u*_2_ and *u*_3_ into four bins each, meaning that there are 16 combinations of these bins. We can calculate the correlations between *u*_1_ and *u*_4_ for each of these bins and plot them as a surface, as in Fig. 8.

The empirical conditional expectation data are not inconsistent with the conditional correlation being a smooth function. The use of a smooth curve to represent the conditional correlation is a possible way of compressing the data more compactly.

The fitted Lagrange multipliers for the two conditional copulas in tree 2, given the binning of *u*_2_ and *u*_3_, respectively, are given in Table IV.

**Table IV:** The Lagrange Multipliers for the Two Conditional Copulas in the Second Tree of the Vine

Copula Variables	Bin	$\left(\alpha_{1},\alpha_{2},\alpha_{3},\alpha_{4}\right)$	$\left(\lambda_{1},\lambda_{2},\lambda_{3},\lambda_{4}\right)$
$u_{1},u_{3}$	$u_{2}\in$(0,0.25)	(0.119,0.071,0.065,0.041)	(48.03,−48.67,−51.07,53.69)
$u_{1},u_{3}$	$u_{2}\in$(0.25,0.5)	(0.188, 0.116, 0.100, 0.063)	(24.57,−21.39,−27.69,25.28)
$u_{1},u_{3}$	$u_{2}\in$(0.5,0.75)	(0.309, 0.215, 0.207, 0.145)	(12.98,−10.54,−9.12,9.71)
$u_{1},u_{3}$	$u_{2}\in$(0.75,1)	(0.488, 0.372, 0.400, 0.307)	(81.61,−76.40,−72.33,69.16)
$u_{2},u_{4}$	$u_{3}\in$(0,0.25)	(0.205, 0.146, 0.123, 0.089)	(49.86,−50.31,−51.80,52.59)
$u_{2},u_{4}$	$u_{3}\in$(0.25,0.5)	(0.216, 0.140, 0.126, 0.083)	(37.30,−35.93,−40.25,39.04)
$u_{2},u_{4}$	$u_{3}\in$(0.5,0.75)	(0.282, 0.185, 0.197, 0.131)	(59.97,−61.00,−57.31,59.77)
$u_{2},u_{4}$	$u_{3}\in$(0.75,1)	(0.307, 0.204, 0.233, 0.157)	(63.21,−64.21,−58.57,60.01)

We fit the minimum information copulas for the different bins defined in the third tree, between *u*_1_ and *u*_4_ given *u*_2_ and *u*_3_. The resulting Lagrange multipliers are found as previously. Details are omitted. This fully defines the vine.

#### 5.2.1. Comparison of Models Using Simulation

To compare how well the two methods considered recover the structures within the data, we simulate from each model.

In the case of the minimum information vine, sampling from the constructed distribution can be carried out using the cumulative approach^14^ (also known as conditional sampling and the inverse of the Rosenblatt transform). The sampling strategy is as follows: sample four independent variables distributed uniformly on interval [0, 1], denoted by $W_{1},W_{2},W_{3},W_{4}$, and calculate values of correlated variables $X_{1},X_{2},X_{3},X_{4}$ by taking $x_{1}=w_{1}$, $x_{2}=F^{-1}\left(w_{2}|x_{1}\right)$, $x_{3}=F^{-1}\left(w_{3}|x_{1},x_{2}\right)$, and $x_{4}=F^{-1}\left(w_{4}|x_{1},x_{2},x_{3}\right)$, where $x_{i}$ and $w_{i}$ are realization values of $X_{i}$ and $W_{i}$, respectively. Pseudo‐code for the sampling, including the binning, is given in the Supporting Information.

Initially, we consider the unconditional correlations in the data that were given in Equation (6). Taking 1,000 samples from the Gaussian copula results in estimates of these correlations being $\rho_{12}=0.619,\rho_{13}=0.341,\rho_{14}=-0.092,\rho_{23}=0.368,\rho_{24}=0.010,$ and $\rho_{34}=-0.090$. A simulation of 1,000 samples from the minimum information vine gives estimates of $\rho_{12}=0.611,\rho_{13}=0.364,\rho_{14}=-0.161,\rho_{23}=0.446,\rho_{24}=-0.098,$ and $\rho_{34}=-0.118$.

Both methods reproduce the overall correlation structure fairly well. However, we saw from Fig. 8 that the conditional correlations between the exchange rates were nonconstant. To see whether our two models are capturing these dependencies, we investigate using cobweb plots.

**Figure 9:**
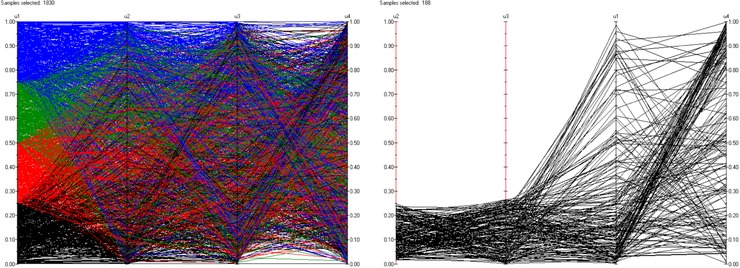
Cobweb plots for all of the data values (left‐hand side) and for $u_{1},u_{4}$ conditional on $u_{2}\in \left[0.75,1\right),u_{3}\in \left[0,0.25\right)$ (right‐hand side).

Initially, we consider the data. On the left‐hand side of Fig. 9 we give a cobweb plot of the four uniform variables for all of the observed time points. We see the overall shape of the distribution. On the right‐hand side of the figure, we see the conditional relationship between $u_{1},u_{4}$ conditional on $u_{2}\in \left[0,0.25\right),u_{3}\in \left[0,0.25\right)$. This relationship is fairly strong. For more information on cobweb plots, see Ref. 44.

**Figure 10:**
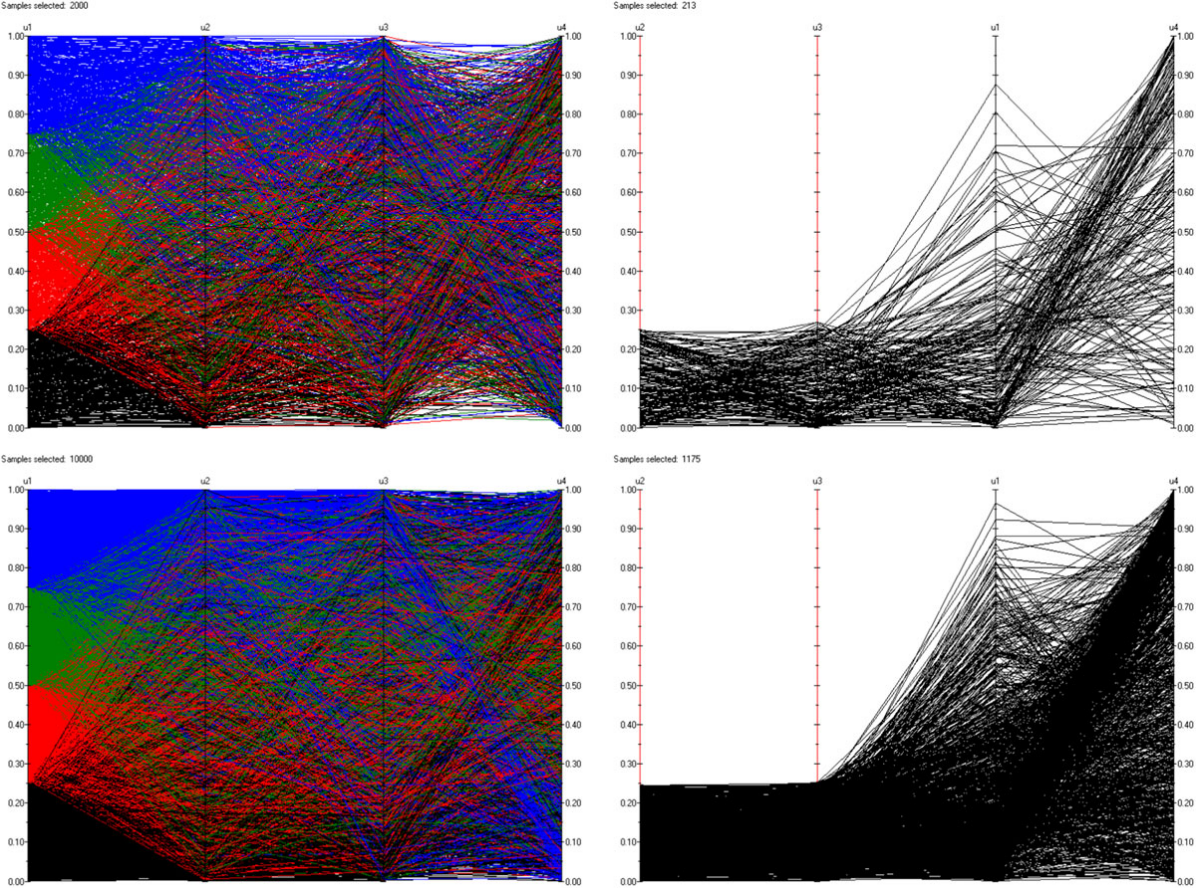
Cobweb plots based on simulated data for the Gaussian copula (top row) and for the minimum information vine (bottom row).

Fig. 10 gives the same two cobweb plots for the Gaussian copula, in the top row, and the minimum information vine, in the bottom row. Both methods are capturing the overall structure of the distribution well. However, when we condition on $u_{2},u_{3}$, the Gaussian copula fails to capture the conditional relationship between $u_{1},u_{4}$. The minimum information vine reproduces a conditional structure that is much closer to that found in the data.

### 5.3. Simulation Study: Tail Dependence

Our general results show that vines formed from minimum information copulas can represent any dependence structure. A much investigated case of dependence in financial and extreme weather risk modeling is tail dependence. In this section, we study tail behavior of the minimum information copula for data simulated from parametric copulas with various tail dependency, including heavy, symmetric, and nonsymmetric tails. Of course, in the real world we compare models to data and not models to models. Nevertheless, the simulation study may provide additional insight. Initially, we utilize scatter plots and Kendall process plots (K‐plots),^45^ which detect bivariate dependence using the ranks of the data.

**Figure 11:**
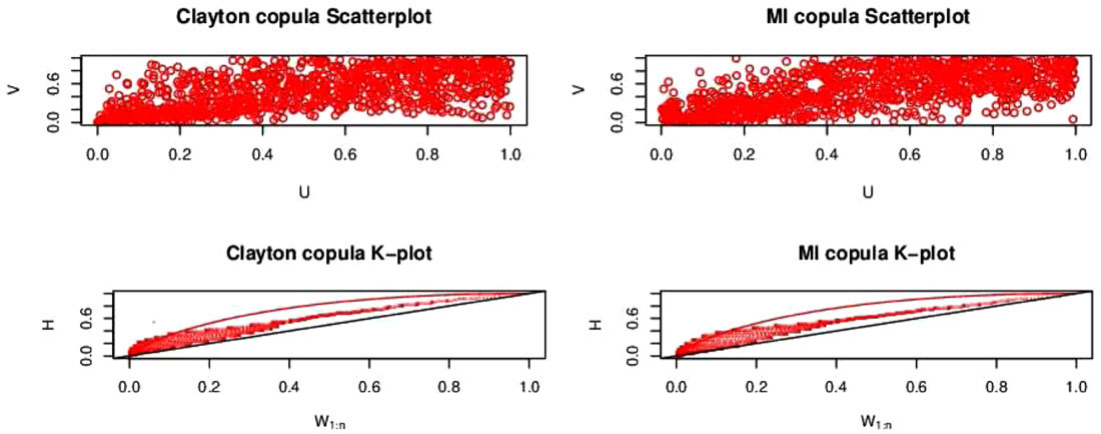
Scatter‐ and K‐plots of the Clayton copula and the fitted minimum information copula.

A simulation study is carried out. We initially investigate data with nonsymmetric tail behavior. It is known that the Clayton and Gumbel copulas have asymmetric tails. The first column of Fig. 11 shows a scatter plot of a random sample taken from a bivariate Clayton copula with parameter θ=1.3979, and the corresponding K‐plot. The second column shows the same plots for a minimum information copula fitted to the sample drawn from the Clayton copula. The basis functions used are $U_{1}U_{2},U_{1}^{2}U_{2},U_{1}^{3}U_{2},U_{1}U_{2}^{2},U_{1}^{4}U_{2}$ and the resulting Lagrange multipliers are 9.02,75.72,−63.81,−20.98,7.54.

From the scatter plots, it appears that the minimum information copula is capturing the general behavior of the Clayton copula well. The upper tail dependency behavior can be observed from the K‐plots and in this case the Clayton and minimum information copula give similar plots. We have repeated the exercise for the Gumbel and *t*‐copulas. The results are given in Appendix B. In all cases, the minimum information copula captures the data from the parametric copula well using the scatter plot and K‐plot.

We extend our investigation to test the ability of minimum information bivariate copulas to capture the upper tail behavior found in *t*, Gumbel, and Tawn copulas. We will investigate the upper tail dependence coefficient. If our minimum information copula can successfully model the behavior in the upper‐right corner of the unit square, then it will also be able to model the behavior in the lower left‐hand corner.

We simulate pairs of 10,000 realizations from each of the parametric copulas identified above for each of 10 sets of parameter values. If θ is the first parameter of each copula and γ is the second parameter, then the values used for the *t*‐copula are θ=(0,0.1,0.2,0.3,0.4,0.5,0.6,0.7,0.8,0.9) (all with γ=4 degrees of freedom) and for the Gumbel and Tawn copulas θ=(1,2,3,4,5,6,7,8,9,10), with γ=1 always for the Tawn copula. The minimum information copula is fit to each of these pairs of observations using six polynomial basis functions of the type given in the examples in this article.

We use a nonparametric estimator^46^, ^47^ of the upper tail coefficient for both the simulated data and the minimum information copula. If pairs of simulated values are $\left(u_{i},v_{i}\right)$ then this is:

$$\begin{array}{ccc} {\hat{\lambda }}_{U} & = & 2-2\exp\left[\frac{1}{n}\sum_{i=1}^{n}\log\left\{\sqrt{\log\left(\frac{1}{u_{i}}\right)\log\left(\frac{1}{v_{i}}\right)}/\right.\right.\\ & & \left.\left.\log\left(\frac{1}{\max{\left(u_{i},v_{i}\right)}^{2}}\right)\right\}\right]. \end{array}$$

**Figure 12:**
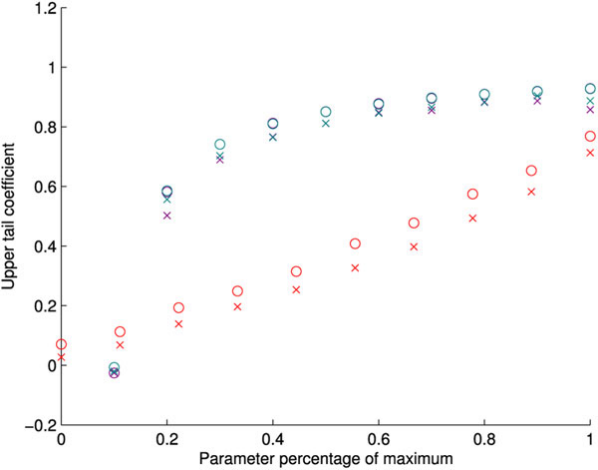
Comparison of upper tail dependence coefficient for simulated values from the *t* (green), Gumbel (purple), and Tawn (gray) copulas and the minimum information copula. The circles represent the parametric copula and the crosses the minimum information copula in each case.

We display the results of the simulation in Fig. 12. The different colors represent the simulated data from the different parametric families. The circles denote the upper tail coefficient for the simulated values and the crosses represent the coefficient for the minimum information copula fitted to the simulated data. In all cases, the estimated values for the simulated data are close to the theoretical values for the parametric copula. On the *x*‐axis, the parameter values from each of the copulas have been scaled to be a percentage of their maximum value in the simulation.

We see that the minimum information copula can model from weak to strong upper tail dependence in all of the parametric copulas chosen. This suggests it is suitable for modeling situations that display tail dependence. Given the basis functions used, however, the minimum information copula tends to underestimate the tail dependence coefficient slightly for all copulas over the ranges of parameter values. This could be improved by choosing basis functions that are concentrated in the top right‐hand corner of the unit square. Future work will address this.

**Figure 13:**
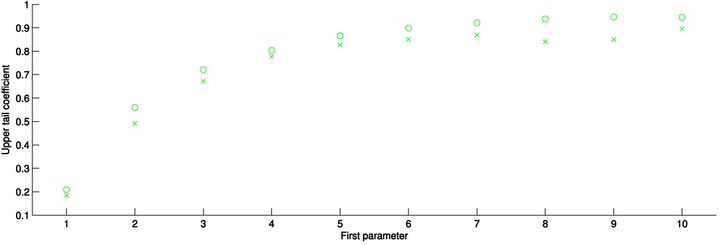
Comparison of upper tail dependence coefficient for simulated values from Gumbel (green) copula and the minimum information D‐vine. The circles represent the parametric copula and the crosses the minimum information vine in each case.

We consider tail‐dependence in the multivariate case. To do so, we simulate tail dependent data in three dimensions $\left(u_{1},u_{2},u_{3}\right)$, fit a D‐vine to the data with bivariate minimum information copulas, and investigate its ability to capture the conditional dependence between $\left(u_{1},u_{3}\right)$ given *u*_2_. We do not need to consider Gaussian or *t*‐copulas as they are closed under bivariate marginalization. We consider the Gumbel copula. The empirical upper tail coefficients from varying parameter values and the estimated upper tail coefficients from the minimum information vine are given in Fig. 13, In each case 10,000 simulations were used.

We see a similar pattern to Fig. 12, with the minimum information approach able to capture high tail dependence between the variables but having a small downward bias in its estimates.

### 5.4. Discussion

#### 5.4.1. Sources of Error

The method we have used has the theoretical property that it can be used to build arbitrarily good approximations to the original distribution. There are several sources of potential error in this approximation that we briefly discuss. One is the choice of base where it is convenient to take a low number of functions $h_{i}$. Using three terms (u.v, $u^{2}v$, $uv^{2}$) rather than one (uv), we are able to generate asymmetric copulas, which has value in modeling general data sets. The use of large numbers of functions does give more accuracy, at the cost of extra computation at the construction stage but at no extra cost at the sampling stage. The second general source of error arises from discretization. Discretization occurs during the construction stage when we have to create data bins with which to calculate conditional expectations from the original data. There is a tradeoff, as with higher numbers of bins we have fewer data and noisier estimates of the conditional expectations. In our sampling, we simply used conditional expectations based on the bins, although we could have used them to estimate continuous functions. If we had used a joint normal distribution, then our implicit assumption would have been that these conditional expectations were all constant. We expect the error arising from this discretization to be the main source of errors overall. A final source of discretization error arises in the level of discretization in the $D_{1}AD_{2}$ algorithm. The algorithm works more slowly when using more points, but gives more accuracy. This becomes more important when the distribution being modeled is far away from uniform.

#### 5.4.2. Vine Structure

We remark on the importance of vine structure. Any regular vine structure can be used to approximate any distribution. Many vine structures are available (see, for example, Kurowica and Cooke^14^), and even with a given vine structure we could permute the variables so that different conditional copulas were being considered in the construction. In view of the above discussion about error, we believe that it makes sense to consider different vine structures, and that a convenient measure of usefulness is the degree of complexity of the conditional expectation functions, which would ideally be constant or simple linear functions.

## 6. CONCLUSION

We present a novel method to approximate a multivariate distribution by any vine structure to any degree of approximation. We have operationalized the theoretical approximation results using minimum information copulas that can be specified to any required degree of precision based on the data available. We have shown rigorously that good approximation “locally” guarantees good approximation globally. This approximation allows the use of a fixed finite‐dimensional family of copulas to be used in a vine construction, with the promise of a uniform level of approximation. That is, we can use the same bases to approximate each copula in each tree of the corresponding vine.

While the choice of vine structure imposes no restrictions on the underlying joint probability distribution it represents, the fact that we only use finite parameter families of copulas means that not every distribution is well represented, and that the choice of vine structure could be significant in closely matching a distribution while still using a simple family of copulas.

Any functions can be used to create the minimum information copulas used here, and in some applications it may be natural to use functions that are themselves computed in computer codes. Because of the frequent evaluation calls needed to determine the min inf distribution, it then makes sense to use emulators or Kriging models as a way to speed up the computations.

Finally, the methods used here generalize well‐known methods such as *Normal to Anything*, used in simulation and decision analysis. This generalization provides us two main advantages: natural ways to generate asymmetric copulas, and simple ways to specify nonconstant conditional correlations (or other moments). Our methods provide a flexible methodology that may be adapted (through the choice of functions $h_{i}$ and the choices about conditional expectations) to produce models taking account of naturally available information at an appropriate level of complexity for the modeling problem.

## Supporting information


**Algorithm 1** To approximate the joint density between two variables of interest, *X* and *Y*, using a minimally informative copula.
**Algorithm 2** To find the log‐likelihood of a minimally informative copula between *X* and *Y* given data $x_{1},...,x_{n}$ and $y_{1},...,y_{n}$.
**Algorithm 3** Calculates the conditional distribution (*Fc*) or density (*fc*) on a grid of points from joint density *f*.
**Algorithm 4** Algorithm to simulate from a 4‐dimensional D‐vine in ($X_{1},X_{2},X_{3},X_{4}$) given uniform marginals and minimum information copulas.

## Figures and Tables

**Figure B.1:**
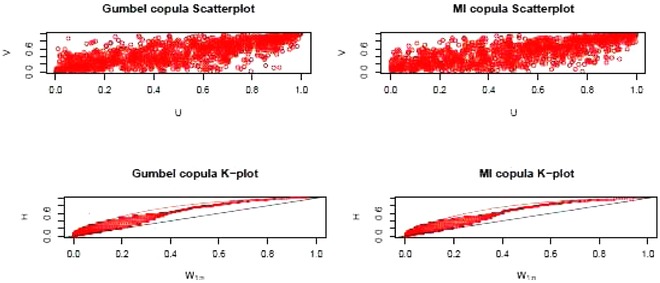
Scatter‐ and K‐plots of the Gumbel copula and the fitted minimum information copula.

**Figure B.2:**
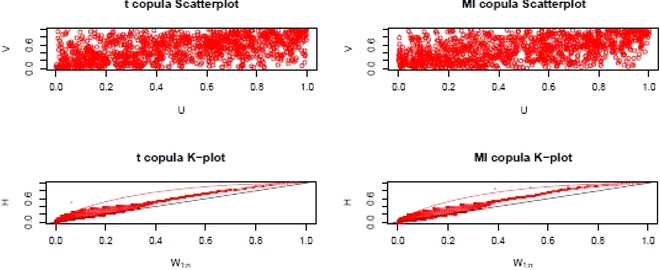
Scatter‐ and K‐plots of the *t*‐copula and the fitted minimum information copula.

## Appendix A

### A.1 Proof of Theorem 2

We have assumed that our multivariate density $f_{1...n}$ is a continuous function defined on [0, 1]^*n*^. Since all marginal densities $f_{i_{1}...i_{r}}$ are obtained by integrating out variables from $f_{1...n}$, it is clear that:

$$|f_{i_{1}...i_{r}}\left(x_{i_{1}},...,x_{i_{r}}\right)\left|\leq \sup\right|f_{1...n}\left(x_{1},...,x_{n}\right)|,$$

where the sup is taken over the variables $x_{i}$ ($i\neq i_{1},...,i_{r}$). Hence,

$$\begin{array}{ccc} |f_{i|i_{1}...i_{r}}\left(x_{i}|x_{i_{1}},...,x_{i_{r}}\right)| & = & |\frac{f_{ii_{1}...i_{r}}\left(x_{i}x_{i_{1}},...,x_{i_{r}}\right)}{f_{i_{1}...i_{r}}\left(x_{i_{1}},...,x_{i_{r}}\right)}\\ & & |\leq ||f||/\alpha , \end{array}$$

where α>0 is a lower bound on the values taken by *f*. This shows that there is a point‐wise bound for all the functions in M(f).

In order to show equicontinuity, we first note that each function $f_{i_{1}...i_{r}}$ is uniformly continuous. Since there are only a finite number of such functions, we can always ensure that given ε>0 there is a δ>0 such that for any $i_{1}...i_{r}$ if

$$|\left(x_{i_{1}},...,x_{i_{r}}\right)-\left(y_{i_{1}},...,y_{i_{r}}\right)|<\delta ,$$

then

$$|f_{i_{1}...i_{r}}\left(x_{i_{1}},...,x_{i_{r}}\right)-f_{i_{1}...i_{r}}\left(y_{i_{1}},...,y_{i_{r}}\right)|<\varepsilon .\alpha .$$

Hence if $|x_{i}-y_{i}|<\delta$, then

$$|f_{i|i_{1}...i_{r}}\left(x_{i}|x_{i_{1}},...,x_{i_{r}}\right)-f_{i|i_{1}...i_{r}}\left(y_{i}|x_{i_{1}},...,x_{i_{r}}\right)|\leq$$

$$|f_{ii_{1}...i_{r}}\left(x_{i},x_{i_{1}},...,x_{i_{r}}\right)\;-\;f_{ii_{1}...i_{r}}\left(y_{i},x_{i_{1}},...,x_{i_{r}}\right)|/\alpha \;\leq \;\varepsilon ,$$

and

$$|\frac{d}{d_{x_{i_{k}}}}f_{i_{1}...i_{r}}\left(x_{i_{1}},...,x_{i_{r}}\right)|\leq \sup$$

so that M(f) must also be an equicontinous family. A similar argument shows that B(f) is an equicontinous family.

### A.2 Proof of Theorem 3

For any element $c_{ij|i_{1}...i_{r}}$ of C(f), we have

$$\begin{array}{ccc} & & c_{ij|i_{1}...i_{r}}\left(u_{i},u_{j}|x_{i_{1}}...x_{i_{r}}\right)\\ & & =\frac{f_{ij|i_{1}...i_{r}}\left(x_{i},x_{j}|x_{i_{1}}...x_{i_{r}}\right)}{f_{i|i_{1}...i_{r}}\left(x_{i}|x_{i_{1}}...x_{i_{r}}\right)f_{j|i_{1}...i_{r}}\left(x_{j}|x_{i_{1}}...x_{i_{r}}\right)}. \end{array}$$

Hence if we take a sequence of elements in C(f), then there are corresponding sequences of elements of M(f) and B(f). Since M(f) is relatively compact there must be a convergent subsequence, and looking along that same subsequence there must be a subsequence of that for which the corresponding functions in B(f) converge. Now, along this subsequence the right‐hand side of the above expression converges, so the elements of C(f) on this same sequence must converge (and to the same thing). In particular, there is a convergent subsequence. Hence, C(f) is relatively compact.

### A.3 Proof of Lemma 1

We show that by taking *f* sufficiently close to *g* one can ensure that the reweighting functions for *f* are close to 1. This then implies that C(f) is close to *g*.

Without loss of generality we can assume that *f* is normalized. The proof uses the fact that we can use the Borwein‐Lewis‐Nussbaum approach to find functions $d_{1f}\left(u\right)$ and $d_{2f}\left(v\right)$ such that $d_{1f}.d_{2f}.f$ has uniform marginals. Such functions $d_{1g}$ and $d_{2g}$ exist also for *g* but are constant, $d_{1g}\left(u\right)=d_{2g}\left(v\right)=1$, because *g* is already a copula. As discussed above, these reweighting functions are fixed points of a functional that is a contraction mapping when using the Hilbert metric D on the appropriate space of pairs of functions $\left(d_{1},d_{2}\right)$.

We denote by $L_{f}$ the functional associated to *f*. Since this is a contraction mapping there exists a $\lambda_{f}\in \left(0,1\right)$ such that

$$D\left(L_{f}\left(a,b\right),L_{f}\left(c,d\right)\right)<\lambda_{f}D\left(\left(a,b\right),\left(c,d\right)\right).$$

If we set $a_{0}=1$, $b_{0}=1$, and $\left(a_{n+1},b_{n+1}\right)=L_{f}\left(a_{n},b_{n}\right)$, then we have convergence to the required pair of functions $\left(d_{1f},d_{2f}\right)$ that reweight *f* to become a copula.

Now, by choosing *f* close enough to *g* we can ensure two things. First, that the contraction rate associated to $L_{f}$ is close to that of $L_{g}$, in particular less than some chosen λ<1. Second, we can ensure that

$$D\left(L_{g}\left(1,1\right),L_{f}\left(1,1\right)\right)=D\left(\left(1,1\right),L_{f}\left(1,1\right)\right)$$

is as small as required. This implies that

$$\begin{array}{ccc} D((1,1),(a,b)) & \leq & \sum_{n=0}^{\infty }D\left(\left(a_{n},b_{n}\right),\left(a_{n+1},b_{n+1}\right)\right)\\ & \leq & D\left(\left(a_{0},b_{0}\right),\left(a_{1},b_{1}\right)\right)\sum_{n=0}^{\infty }\lambda^{n}\\ & = & \frac{D(\left(1,1\right),L_{f}\left(1,1\right))}{1-\lambda }. \end{array}$$

Hence, the reweighting functions for *f* are close to the identity, and so C(f) is close to *g*.
