# Supplementary material for: Approximate Uncertainty Modeling in Risk Analysis with Vine Copulas
Source: Risk Anal. 2015 Sep 2;36(4):792–815. doi: 10.1111/risa.12471 (PMC4989465; doi:10.1111/risa.12471)
Supplement: Supplementary file 1 — Algorithm 1 To approximate the joint density between two variables of interest, X and Y, using a minimally informative copula. Algorithm 2 To find the log‐likelihood of a minimally informative copula between X and Y given data x1,...,xn and y1,...,yn. Algorithm 3 Calculates the conditional distribution (Fc) or density (fc) on a grid of points from joint density f. Algorithm 4 Algorithm to simulate from a 4‐dimensional D‐vine in (X1,X2,X3,X4) given uniform marginals and minimum information copulas. [file RISA-36-792-s001.pdf]

Supplementary material to: Approximate Uncertainty Modelling in Risk Analysis with Vine Copulas

July 10, 2014

1 Pseudo-code

In this section of the supplementary material we give pseudo code associated with Example 3. Algorithm 1 gives a procedure for approximating the minimum information copula between two random quantities. Algorithm 2 then shows how to calculate the log-likelihood of the copula in this case.

In terms of the simulation we need some generic functions which will be used in several steps. The first, $\mathrm{Cond}(x, f, \boldsymbol{u})$, finds the distribution function or density conditional on the value x from a joint density f over a grid of points in the unit square defined by vector $\boldsymbol{u} = (u_1, \ldots, u_g)$. This is given in Algorithm 3. The second $Bin_m(x, f)$ selects the correct conditional copula density from m candidates f based on which bin x falls into.

Given these functions, the simulation takes a similar form to that given in Kurowicka and Cooke (2006). This is set out in Algorithm 4. The joint minimum information copula densities, which are inputs to the algorithm, are evaluated on the discretized grid of points.

Algorithm 1 To approximate the joint density between two variables of interest, X and Y, using a minimally informative copula.

Require: $m =$ number of the bases denoted by $h_1(X,Y), \ldots, h_m(X,Y)$
α_l: the l'th required expectation
$\lambda_0 = (\lambda_{01}, \ldots, \lambda_{0m})$ Lagrange multipliers initial values (often set to 0)
$k =$ number of the discretization points per dimension
$u = \frac{1}{2k} : \frac{1}{k} : (1 - \frac{1}{2k})$ create discrete points in interval (0, 1)
$x_p = F_X^{-1}(u_f)$ is a $k \times 1$ vector of the u_f percentiles of the values in x
$y_p = F_Y^{-1}(u_f)$ is a $k \times 1$ vector of the u_f percentiles of the values in y
$V_l = h_l(x_p, y_p)$ is a $k \times k$ matrix corresponding to the values of the l_{th} function calculated at x_p, y_p
for $i = 1 : k$ **do**
  for $j = 1 : k$ **do**
    $A(i,j) = \exp(\sum_{l=1}^{m} \lambda_l V_l(i,j)), \quad i,j = 1, \ldots, k$ is a $k \times k$ kernel matrix
  end for
end for
Require: k_0 a suitably large enough iterative number
$D_1 = \mathbf{1}_{k \times 1}$
$D_2 = D_1$
loop
  $D_1 = k/A^T D_2$
  $D_2 = k/AD_1$
end loop
$\mathbf{B}$=$D_2 D_1^T A$ minimally informative copula density
for $l = 1 : m$ **do**
  $L_l = \frac{1}{k^2} \sum_{j=1}^{k} \sum_{i=1}^{k} \mathbf{B}(i,j) V_l(i,j) - \alpha_l$
end for
$\lambda = \arg\min_\lambda (\sum_{l=1}^{m} L_l^2)$

Algorithm 2 To find the log-likelihood of a minimally informative copula between X and Y given data $x_1, \ldots, x_n$ and $y_1, \ldots, y_n$.

```
a = 1/2k
for i = 1 : n do
  for j = 1 : k do
    if |u_j − F_X(x_i)| < a then
      F_X(x_i) ← u_j
      Index1 = j
    end if
  end for
  I_1(i) = Index1;
end for
for i = 1 : n do
  for j = 1 : k do
    if |u_j − F_Y(y_i)| < a then
      F_Y(y_i) ← u_j
      Index2 = j
    end if
  end for
  I_2(i) = Index2;
end for
for i = 1 : n do
  fc(i) = log(B(I_1(i), I_2(i)));
end for
log-likelihood= sum(fc)
```

Algorithm 3 Calculates the conditional distribution (Fc) or density (fc) on a grid of points from joint density f

for $j = 1 : g$ **do**
  if $x > u_j$ **then**
    index= j
  end if
end for
$w_1 = (u_{index+1} - x)/(u_{index+1} - u_{index})$
$w_2 = 1 - w_1$
for $j = 1 : g$ **do**
  $fc_j = w_1 f_{index,j} + w_2 f_{index+1,j}$
end for
$fc = fc/\sum_j fc_j$
$Fc_1 = fc_1$
for $j = 2 : g$ **do**
  $Fc_j = Fc_{j-1} + fc_j$
end for

Algorithm 4 Algorithm to simulate from a 4 dimensional D-vine in (X_1, X_2, X_3, X_4) given uniform marginals and minimum information copulas.

Sample $u_1, \ldots, u_4$ where $u_i \sim U(0,1)$.
$x_1 = u_1$
$F_{2|1}$ =Cond(x_1, f_{12}, G)
$x_2 = F_{2|1}^{-1}(u_2)$
$f_{1|2}$ =Cond(x_2, f_{12}, G)
$f_{3|2}$ =Cond(x_2, f_{23}, G)
$f'_{13|2}$ =Bin$_4(x_2, f_{13|2})$
$f_{13|2} = f_{1|2} f_{3|2} f'_{13|2}$
$F_{3|12}$ =Cond$(x_1, f_{13|2}, G)$
$x_3 = F_{3|12}^{-1}(u_3)$
$f_{4|3}$ =Cond(x_3, f_{34}, G)
$f_{2|3}$ =Cond(x_3, f_{23}, G)
$f'_{24|3}$ =Bin$_4(x_3, f_{24|3})$
$f_{24|3} = f_{2|3} f_{4|3} f'_{24|3}$
$f_{4|23}$ =Cond$(x_2, f_{24|3}, G)$
$f_{1|23}$ =Cond$(x_3, f_{13|2}, G)$
$f'_{14|23}$ =Bin$_{16}([x_2, x_3], f_{14|23})$
$f_{14|23} = f_{1|23} f_{4|23} f'_{14|23}$
$F_{4|123}$ =Cond$(x_1, f_{14|23}, G)$
$x_4 = F_{4|123}^{-1}$

References

Kurowicka, D., and Cooke. R. (2006). *Uncertainty Analysis with High Dimensional Dependence Modelling.* John Wiley.
